# Determination of tyrosinase-cyanidin-3-*O*-glucoside and (−/+)-catechin binding modes reveal mechanistic differences in tyrosinase inhibition

**DOI:** 10.1038/s41598-021-03569-1

**Published:** 2021-12-30

**Authors:** Kyung Eun Lee, Shiv Bharadwaj, Amaresh Kumar Sahoo, Umesh Yadava, Sang Gu Kang

**Affiliations:** 1 Department of Biotechnology, Institute of Biotechnology, College of Life and Applied Sciences, Yeungnam University, 280 Daehak-Ro, Gyeongsan, 38541 Gyeongbuk Korea; 2 Department of Applied Sciences, Indian Institute of Information Technology Allahabad, Allahabad, 211015 Uttar Pradesh India; 3 Department of Physics, Deen Dayal Upadhyay Gorakhpur University, Gorakhpur, India; 4 Stemforce, 313 Institute of Industrial Technology, Yeungnam University, 280 Daehak-Ro, Gyeongsan, 38541 Gyeongbuk Korea; 5 Present Address: Laboratory of Ligand Engineering, Institute of Biotechnology of the Czech Academy of Sciences, BIOCEV Research Center, Vestec, Czech Republic

**Keywords:** Virtual screening, Biochemistry, Computational biology and bioinformatics, Drug discovery, Biophysics, Computational biophysics

## Abstract

Tyrosinase, exquisitely catalyzes the phenolic compounds into brown or black pigment, inhibition is used as a treatment for dermatological or neurodegenerative disorders. Natural products, such as cyanidin-3-*O*-glucoside and (−/+)-catechin, are considered safe and non-toxic food additives in tyrosinase inhibition but their ambiguous inhibitory mechanism against tyrosinase is still elusive. Thus, we presented the mechanistic insights into tyrosinase with cyanidin-3-*O*-glucoside and (−/+)-catechin using computational simulations and in vitro assessment. Initial molecular docking results predicted ideal docked poses (− 9.346 to − 5.795 kcal/mol) for tyrosinase with selected flavonoids. Furthermore, 100 ns molecular dynamics simulations and post-simulation analysis of docked poses established their stability and oxidation of flavonoids as substrate by tyrosinase. Particularly, metal chelation via catechol group linked with the free 3-OH group on the unconjugated dihydropyran heterocycle chain was elucidated to contribute to tyrosinase inhibition by (−/+)-catechin against cyanidin-3-*O*-glucoside. Also, predicted binding free energy using molecular mechanics/generalized Born surface area for each docked pose was consistent with in vitro enzyme inhibition for both mushroom and murine tyrosinases. Conclusively, (−/+)-catechin was observed for substantial tyrosinase inhibition and advocated for further investigation for drug development against tyrosinase-associated diseases.

## Introduction

Melanin synthesis is a sequence of convoluted biochemical events and involves tyrosinase family proteins such as tyrosinase, tyrosinase-related protein-1 (TRP-1), and TRP-2^1,2^. Tyrosinase (EC 1.14.18.1), also termed polyphenol oxidase (PPO)—a copper-containing metalloprotein is ample in bacteria, fungi, mammals, and plants^3,4^, and their active sites are exceedingly conserved between the diverse species^5–8^. Tyrosinase exquisitely catalyzes two distinct reactions critical for the melanin synthesis: the hydroxylation of l-tyrosine (hydroxylate monophenols) to 3,4-dihydroxyphenylalanine (l-DOPA or (*o*)ortho-diphenols) via a process named tyrosinase monophenolase activity and subsequently proceeds to process termed diphenolase activity, which causes oxidation of *o*-diphenols (l-DOPA) into *o*-quinones (DOPA quinone)^9–11^. The generated reactive quinones demonstrate instant polymerization to produce high molecular weight melanin nonenzymatically^12,13^. Notably, tyrosinase possesses two copper ions, i.e., CuA and CuB—coordinate with six histidine (His) residues in the conserved catalytic pocket^14,15^, and are critically required to exhibit both types of enzymatic activities^6,16^.

In mammals, tyrosinase organizes the melanin synthesis to defend the skin from harmful effects of ultraviolet (UV) radiations^17^, while hyperpigmentation disorders noted to promote freckles, melisma, pigmentation, petaloid actinic tanning, solar lentigo, and senile lentigines malignant melanoma^18–20^. Tyrosinase also prompts the oxidation of dopamine to form melanin in the brain; and hence, linked with the pathogenesis of neurodegenerative disorders, including Parkinson’s disease^21–23^. Additionally, tyrosinase has been suggested to contribute on the onset of autoimmune diseases^24^. Therefore, tyrosinase inhibitors are categorically called for by the cosmetics and pharmaceutical industries^11,23,25,26^.

Numerous natural products, particularly polyphenols and plant-derived extracts, are well-recognized to inhibit tyrosinase enzyme^27–29^. Among the various natural products, ubiquitous hydroxylated flavonoids have been documented as a potent inhibitor of tyrosinase due to their structural similarities with tyrosinase substrates, such as l-tyrosine and l-DOPA, and substantial antioxidant properties^11,29–31^. Moreover, many common polyphenols are known to inhibit tyrosinase by acting as “alternative substrates, such as catechins, caffeic acid, and tyrosol^32–34^. However, the presence of such compounds in the extract or fraction during Bioactivity-guided fractionation (BGF) using mushroom tyrosinase (mh-Tyr) was elucidated to interfere with the enzyme inhibition assay due to the production of similar by-product that exhibit similar maximum light absorbance as those of the tyrosinase substrates, viz. l-tyrosine and l-DOPA^29^. Therefore, it is apparent that polyphenolic compounds, such as flavonoids, interfere with the absorb light in spectroscopic methods to produce pseudo-mh-Tyr inhibition results^29^. Interestingly, among several natural products, cyanidin-3-*O*-glucoside and catechins were studied and reported as mh-Tyr inhibitors using spectroscopic methods, recently reviewed elsewhere^35^. Based on these observations, it is essential to elucidate the subtle mechanistic interactions between the tyrosinase and flavonoids to provide direct evidence of the later inhibition, which is still unresolved.

**Figure 1:**
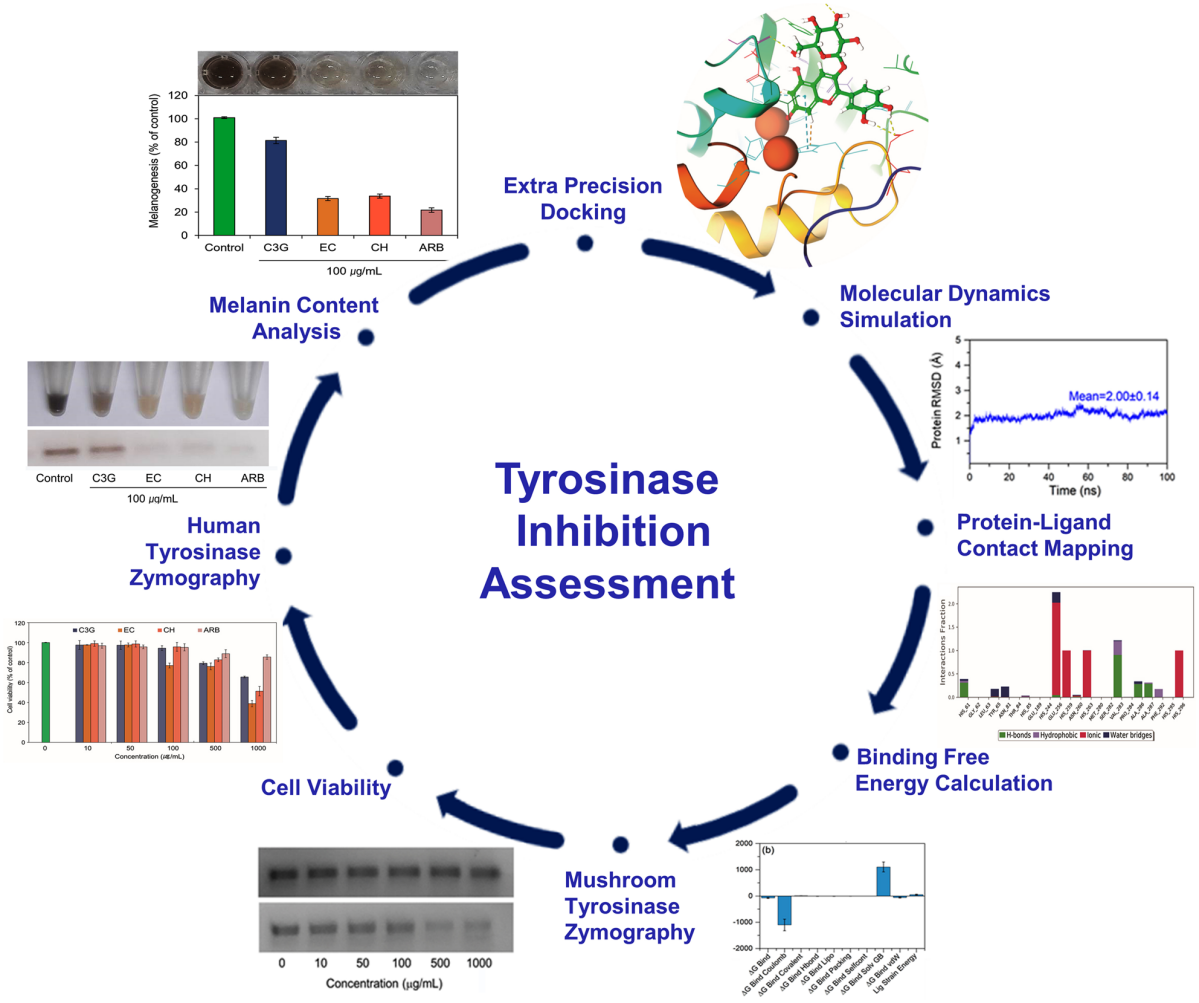
Scheme for the assessment of tyrosinase inhibition by cyanidin-3-*O*-glucoside and (−/+)-catechin using in silico and in vitro methods.

Hence, we present the molecular interactions and binding poses of selected flavonoids (anthocyanidin such as the cyanidin-3-*O*-glucoside and (−/+)-catechins such as (−)-epicatechin and (+)-catechin) in the catalytic pocket of mh-Tyr (in absence of mammalian tyrosinase crystal structure) using computational approaches. Furthermore, to assess the tyrosinase inhibition without the interference of generated byproducts from the chosen flavonoids by tyrosinase, zymography—an electrophoretic method for the detection of hydrolytic enzymes, based on the substrate repertoire of the enzyme was also employed as depicted in Fig. 1.

## Methodology

### Computational analysis

#### Ligands and receptor crystal structure collection

Three-dimensional (3D) structure of selected flavonoids, viz., cyanidin-3-*O*-glucoside (C3G) (CID: 441667), (−)-epicatechin (EC) (CID: 72276), and (+)-catechin (CH) (CID: 9064), and positive control, i.e., arbutin (CID: 440936), were collected from the PubChem database (https://pubchem.ncbi.nlm.nih.gov)^36^. Also, the 3D crystallographic structure of tyrosinase from *Agaricus bisporus* mushroom with a tropolone inhibitor (PDB ID: 2Y9X)^37^ was downloaded from the RCSB protein database (http://www.rcsb.org/)^38^. Moreover, as the catalytic pockets of tyrosinases have been reported to exceedingly conserved across the diverse species^5^ and mammalian tyrosinase crystal structure is not available yet, homology model of human tyrosinase (UniProtKB-P14679) was collected from AlphaFold database (https://alphafold.ebi.ac.uk)^39^ and aligned with the 3D crystallographic structure of mushroom tyrosinase (mh-Tyr) using Superimpose tool in the Maestro v12.6 tool of Schrödinger suite-2020.4^40^. All the 2D and 3D images of both the ligands and receptor were rendered in the free academic version of Maestro v12.6 tool of Schrödinger suite-2020.4^40^.

#### Preparation of ligand and receptor

To perform the molecular docking simulation, 3D structures of the selected ligands, viz. cyanidin-3-*O*-glucoside (C3G), (−)-epicatechin (EC), (+)-catechin (CH), and arbutin (ARB inhibitor), were treated for desalting and tautomer generation, retained with specific chirality (vary other chiral centers), and assigned for metal-binding states by Epik at neutral pH for computation of 32 conformations per ligand using the LigPrep module^41^. Likewise, the crystal structure of mushroom tyrosinase (mh-Tyr), was preprocessed using PRIME tool^42,43^ and protein preparation wizard^44^ under default parameters in the Schrödinger suite-2020.4^45^. Herein, the mh-Tyr crystal structure was also processed by deletion of co-crystallized ligand and water molecules, the addition of polar hydrogen atoms, optimization of hydrogen-bonding network rotation of thiol and hydroxyl hydrogen atoms, tautomerization and protonation states for histidine (His) residue, assignments of Chi ‘flip’ for asparagine (Asn), glutamine (Gln), and His residues, and optimization of hydrogen atoms in distinct species achieved by the Protein preparation wizard. Correspondingly, standard distance-dependent dielectric constant at 2.0 Å, which specifies the small backbone fluctuations and electronic polarization in the protein, and conjugated gradient algorithm were used in the successive enhancement of protein crystal structure, including merging of hydrogen atoms, at root mean square deviation (RMSD) of 0.30 Å under optimized potentials for liquid simulations-3e force field (OPLS-3e) using Protein preparation wizard in the Schrödinger suite-2020.4^45^.

#### Molecular docking and pose analysis

To monitor the binding affinity of selected flavonoids with mh-Tyr, the active residues, viz. His^61^, His^85^, His^259^, Asn^260^, His^263^, Phe^264^, Met^280^, Gly^281^, Phe^292^, Ser^282^, Val^283^, and Ala^286^, and copper ion (Cu^401^) interacting with the co-crystallized tropolone inhibitor in the crystal structure of mh-Tyr^37^ were considered for the screening of selected flavonoids (C3G, EC, and CH) and positive control (ARB inhibitor) using extra precision (XP) docking protocol of GLIDE v8.9 tool under default parameters in the Schrödinger suite-2020.4^46^. Herein, mh-Try structure as receptor was considered as rigid while selected compounds as ligands were allowed to move as flexible entities to discover the most feasible intermolecular interactions in the binding pocket of the receptor. During molecular docking procedure, Coulombic interactions, freezing of rotatable bonds, hydrophobic contacts, intermolecular hydrogen bonds, metal bond formations, polar contacts, the penalty for buried polar groups, van der Waals interactions, solvent (water) desolvation energy, and binding affinity elevating intermolecular contacts formation were allowed in the XP docking scoring protocol^47,48^. Finally, intermolecular contacts formed in the docked complexes were visualized and analyzed using the free academic Maestro v12.6 tool of Schrödinger suite-2020.4^40^. Further, the co-crystalized tropolone inhibitor in the 3D crystallographic structure of tyrosinase from *Agaricus bisporus* mushroom (PDB ID: 2Y9X)^37^ was extracted and re-docked under similar parameters to validate the docking protocol and marked as a reference inhibitor for later in silico analysis.

#### System preparation and explicit molecular dynamics simulation

The best poses of the receptor-ligand docked complexes were collected corresponding to the highest negative docking scores and subjected to 100 ns  classical molecular dynamics (MD) simulation in an explicit solvent under Linux environment on HP Z2 Microtower workstation using the free academic version of Desmond v5.6^49^ module in Maestro-Schrödinger suite 2018-4^50^. Herein, the simulation system was amended with an explicit TIP4P (transferable intermolecular potential-4 point) solvent model, as an orthorhombic box (10 × 10 × 10 Å buffer) followed by the addition of 0.15 M salt to provide a physiological environment using the System Builder tool. Moreover, the complete simulation system was neutralized using counter sodium and chloride ions and placed at 20 Å distance around the docked ligand in the prepared simulation system. Following, the complete system was subjected to minimization under default parameters, viz. maximum iterations of 2000 and 1.0 kcal/mol/Å convergence threshold, using Minimization tool in Desmond module with Maestro-Schrödinger suite 2018–4 interface. Finally, the prepared system for each receptor-ligand complex was allowed for 100 ns MD simulation under default parameters using OPLS-2005 force field with the normal temperature (300 K) and pressure (1 bar), and a total of 10,000 frames were accumulated at successive 10 ps interval with molecular dynamics tool in the free academic Desmond module with Maestro-Schrödinger suite 2018-4 interface. Initially, the re-docked reference complex was subjected to 100 ns MD simulation to validate the simulation parameters in the Desmond module followed by evaluation of docked flavonoids in the catalytic pocket of mh-Tyr.

#### Post-simulation computation

Following 100 ns MD simulation, initially, the last poses were collected from each simulation trajectory and analyzed for the displacement of docked ligand by comparison to the respective initial conformation using superimpose module in the free academic Maestro v12.6 tool of Schrödinger suite-2020.4^40^. Furthermore, each trajectory was statistically analyzed in terms of root-mean-square deviation (RMSD), root-mean-square fluctuation (RMSF), and protein–ligand intermolecular interactions using Simulation Interaction Diagram (SID) module in the free academic version of Desmond-Maestro v11.8 suite^49,50^.

#### Essential dynamics computation

Essential dynamics, as expressed by principal component analysis (PCA), is a statistical method to determine the collective modules of essential fluctuations in the residues of the protein by calculation and diagonalization of the covariance matrix of the carbon-alpha (Cα) atoms^51,52^. Herein, the calculated orthogonal vectors or eigenvectors with the highest eigenvalues are named principal components (PCs). In this study, essential dynamics assessment was performed for each generated MD trajectory using Bio3d package (Released version 2.4-1; http://thegrantlab.org/bio3d/)^51^ under R environment (R version 4.0.4; http://mirror.fcaglp.unlp.edu.ar/CRAN/)^53^. Briefly, all the Cα atoms in the residues of the protein structure present in the 10,000 frames produced by 100 ns MD simulation were aligned to the initial pose. This superimposition was conducted to reduce the root mean square variances between the corresponding residues in the protein structure, and then corresponding PCs were calculated under default parameters using the Bio3d package^51^.

#### Binding free energy calculation

Among the various available approaches for binding free energy predictions, the molecular mechanics generalized Born surface area (MM/GBSA) method has been suggested to provide the rational results^54,55^. Therefore, MM/GBSA method was utilized to evaluate the binding strength of docked flavonoids (C3G, EC, and CH) and ARB inhibitor in the active pocket of the mh-Tyr before (docked poses) and after 100 ns MD simulation (snapshots extracted from the last 10 ns interval). Equations (1)–(4) indicates the mathematical description to compute the binding free energy by MM/GBSA method and respective energy dissociation components.

1 $${\mathrm{\Delta G}}_{Bind}={\mathrm{\Delta G}}_{Com}-\left({\mathrm{\Delta G}}_{Rec }+ {\mathrm{\Delta G}}_{Lig}\right)=\mathrm{\Delta H}-\mathrm{T\Delta S }\approx {\mathrm{\Delta E}}_{MM}+{\mathrm{\Delta G}}_{sol}-\mathrm{T\Delta S}$$

2 $${\mathrm{\Delta E}}_{MM}={\mathrm{\Delta E}}_{Int}+{\mathrm{\Delta E}}_{Ele }+{\mathrm{\Delta E}}_{vdW}$$

3 $${\mathrm{\Delta G}}_{Sol}={\mathrm{\Delta G}}_{GB}+{\mathrm{\Delta G}}_{SA }$$

4 $${\mathrm{\Delta G}}_{SA}= \gamma .\mathrm{SASA}+b$$

In Eq. (1), ΔG_*Bind*_ indicates the binding free energy, ΔG_*Com*_ represents the total free energy in docked receptor-ligand complex, and ΔG_*Rec*_ + ΔG_*Lig*_ depicts the sum of free-state energy of receptor and ligand. Based on the second law of thermodynamics, as mentioned in Eq. (1), binding free energy (Δ*G*_*Bind*_) calculated for the docked receptor-ligand complex can be classified as the total sum of the enthalpy part (Δ*H*) and change of conformational entropy (− TΔ*S*) in the considered system. In this study, the entropy term was neglected due to its excessive computational cost and comparatively low prediction accuracy to the final binding free energy^56,57^. Therefore, the net binding free energy was defined using the total enthalpy in the system and expressed as a summation of total molecular mechanical energy (ΔE_*MM*_) and solvation free energy (ΔG_*Sol*_). Characteristically, ΔE_*MM*_ signifies the assemblage of the intermolecular energies (ΔE_*Int*_), i.e., bond, angle, and dihedral energy, the electrostatic energy (ΔE_*Ele*_), and the van der Waals interaction (ΔE_*vdW*_) as cited in Eq. (2). While electrostatic solvation energy (Δ*G*_*Sol*_) denotes the total sum of polar (ΔG_*GB*_) and nonpolar energy (ΔG_*SA*_) between the continuum solvent and solute in the complete system under consideration as given in Eq. (3). Typically, as shown in Eq. (3-4), the contribution of polar interactions is calculated using the generalized Born (GB) model, and the nonpolar interactions are calculated using the solvent-accessible surface area (SASA)^58^. In Eq. (4), *b* stands for the constant and gamma (*γ*) represents the surface tension parameter for the system and is calculated by measuring the experimental hydration free energy of saturated linear hydrocarbons. In this study, the binding free energy for both docked protein–ligand poses and snapshots mined from 100 ns MD simulation trajectory of respective complexes was computed with default parameters in Prime MM/GBSA module of Maestro-Schrödinger suite 2020.4^43,45^.

## In vitro activity

### Materials and chemicals

In this study, all the chemicals of analytical grade were procured and used in the experimental study. For instance, cyanidin-3-*O*-glucoside (C3G), (−)-epicatechin (EC), and (+)-catechin hydrate (CH), arbutin (ARB inhibitor), *Agaricus bisporus* tyrosinase or mushroom tyrosinase (mh-Tyr), and l-DOPA/l-tyrosine were procured from the Sigma-Aldrich Corporation., St. Louis, MO, USA.

### Mushroom tyrosinase inhibition assay

Mushroom tyrosinase (mh-Tyr) inhibition by the selected flavonoids (C3G, EC, and CH) and positive inhibitor (ARB inhibitor) was monitored using a previously explained method by Maeda et al.^59^ with minor modifications. Briefly, 300 µL reaction mixture was prepared by addition of 200 µL of 0.1 M phosphate buffer (pH 6.5), 40 µL of 1.5 mM l-tyrosine, 40 µL of the selected compounds (10–1000 μg/mL), 20 µL of mh-Tyr (2000 U/mL) solution, and later incubated at 37 °C for 10 min. After that, the total amount of dopachrome produced in the enzyme reaction mixture was determined by absorbance at 490 nm by a microplate reader (Infinite F200, TECAN, Männedorf, Switzerland).

### Mushroom tyrosinase zymography

Mushroom tyrosinase (mh-Tyr) inhibition by the selected flavonoids (C3G, EC, and CH) and positive control (ARB inhibitor) was also elucidated using the zymography method. Briefly, various concentrations (10–1000 μg/mL) of selected compounds were mixed with the mh-Tyr (2000 U/mL) and 5X sample buffer [1.5 M Tris–HCl (pH 6.8), 10% glycerol, and 0.01% bromophenol blue] followed by incubation on the ice for 30 min. After that, each reaction mixture (25 μL) was loaded in 7.5% SDS along with protein marker, and electrophoresis was performed at 4 °C. Next, the gel was washed twice with deionized water and then rinsed with 0.1 M sodium phosphate buffer (PBS) (pH 6.8) for 30 min with gentle shaking at room temperature. Following this, the gel was rinsed twice with deionized water and incubated with 0.01% of l-DOPA at 37 °C for 4 h for the development of dark-brown color bands by the enzymatic activity of the mh-Tyr. Finally, the color bands produced in the gel against each concentration of selected compounds were measured using LabWorks software (UVP, Upland, CA, USA) and used to express the percentage activity of mh-Tyr in reference to control (without any treatment).

### Measurement of cell viability

An MTT assay was conducted to establish the impact of selected flavonoids (C3G, EC, and CH) and positive control (ARB inhibitor) on the murine melanoma cells using CellTiter 96 AQueous One Solution Cell Proliferation Assay Kit (Promega, USA). Herein, murine melanoma cells B16F10 (ATCC, Manassas, VA, USA) culture was maintained in Dulbecco's Modified Eagle Medium (DMEM) (Welgene, Gyeongsan, Gyeongbuk, Korea) containing 10% fetal bovine serum (FBS) (Welgene, Gyeongsan, Gyeongbuk, Korea), and penicillin (100 U/mL)/streptomycin (100 μg/mL) (Welgene, Gyeongsan, Gyeongbuk, Korea) at 37 °C under 5% CO_2_ atmosphere in a CO_2_ cell incubator (NU-4750G, NuAire, Plymouth, MN, USA). To calculate the cell viability, the cultured cells were uniformly distributed (1 × 10^3^ cells/well) in a cell culture plate and incubated for the next 24 h, and subsequently treated with different concentrations (10–1000 μg/mL) of test and control compounds for the next 5 days similar to cell culture conditions. After that, all the culture media was replenished by 100 μL DMEM medium and 20 μL MTS reagent (3-(4,5-dimethylthiazol-2-yl)-5-(3-carboxymethoxyphenyl)-2-(4-sulfophenyl)-2H-tetrazolium inner salt). Finally, the above reaction mixtures were further incubated under dark for 3 h in 5% CO_2_ at 37 °C and then measured for optical density at 490 nm using the microplate reader (Infinite F200, TECAN, Männedorf, Switzerland). Also, a set without treatment was used as a reference control, and percentage cell viability was calculated by applying the Eq. (5).

5 $${\mathrm{Cell}} \, {\mathrm{ viability} }\left(\mathrm{\%}\right)=\left(\frac{{\mathrm{Absorbance}} \, {\mathrm{of}} \, {\mathrm{the}} \, {\mathrm{treated}} \, {\mathrm{group}}}{{\mathrm{Absorbance}} \, {\mathrm{of}} \, {\mathrm{the }} \, {\mathrm{control}} \, {\mathrm{ group}}}\right)\times 100$$

### Murine melanoma cell tyrosinase zymography assay

The flavonoids (C3G, EC, and CH) and positive control (ARB inhibitor) were also monitored for the mammalian tyrosinase inhibition using tyrosinase zymography assay. Herein, 24 h old murine melanoma cell culture was diluted to 1 × 10^4^ cells/mL and treated with the least toxic concentration (μg/mL) of each selected compound. The treated cells were then incubated for the next 5 days, the medium was withdrawn, and cells were rinsed twice with Dulbecco′s Phosphate Buffered Saline (DPBS) (WELGENE, Gyeongsan, Gyeongbuk, Korea). Following, collected cells were dissolved in 200 μL of Protein Extraction Reagent (ThermoFisher Scientific, Waltham, MA, USA) to extract the total cellular protein content. Next, an aliquot of the lysate was used to quantify the protein content using the BCA Protein Assay Kit (ThermoFisher Scientific, Waltham, MA, USA). After that, 60 μg of protein was mixed with sampling buffer and resolved on 7.5% SDS–polyacrylamide gel electrophoresis (PAGE). Then, the gel was washed twice with deionized water followed by rinsing in 0.1 M DPBS (pH 6.8) for 30 min with gentle shaking at room temperature. Following, the gel was again rinsed twice with water and incubated in 0.01% l-DOPA staining solution in the dark for 4 h at 37 °C. The activity of cellular tyrosinase was then visualized in the gel as dark melanin-containing bands and quantified in terms of color intensity using the LabWorks program (UVP, Upland, CA, USA) for the percentage mammalian tyrosinase activity with reference to control (without treatment).

### Determination of melanin content

The total concentration of melanin produced by the treated cells was calculated as a previously reported method by Tsuboi et al.^60^ with minor modifications. In brief, 24 h old murine melanoma B16F10 cell culture was uniformly distributed (1 × 10^4^ cells/mL) in the cell culture plates and amended with the least toxic concentration (μg/mL) of each selected compound, incubated under culture conditions for next 5 days. Next, the culture medium was discarded while collected cells were gently rinsed twice with 0.1 M DPBS (pH 6.8). Following, the cell pellets, containing a known number of cells (~ 1 × 10^6^ cells/mL), were dissolved in 1 mL reagent: 1 N sodium hydroxide (NaOH) and 10% DMSO, and boiled at 60 °C for 30 min. Finally, the optical density of the lysate was determined at 490 nm using the microplate reader (Infinite F200, TECAN, Männedorf, Switzerland) to calculate the total melanin content in the treated cells in reference to control (without treatment).

### Statistical analysis

In this study, all the tests were conducted in triplicates and findings were given as the average of experiments with standard deviation (SD). Moreover, the P-value (< 0.05) was studied to indicate the intergroup substantial differences and concluded by one-way analysis of variance (ANOVA) with Fisher’s protected least significant difference (PLSD) test in StatView software (Version 5.0.1., SAS Institute Inc., Cary, NC, USA).

## Results

### Molecular docking and intermolecular interaction analysis

Tyrosinase (EC 1.14.18.1) is an enzyme that shows dual activities, i.e., monooxygenase and oxidase function, which occurs by the dioxygen binding with the two copper atoms, viz. CuA and CuB, positioned in the catalytic pocket^9,16^. Several X-ray crystal structures of tyrosinase have been established from different species, including fungi and bacteria; however, mammalian or human-tyrosinase 3D crystal structure is not yet available. Besides, tyrosinase from bacterial and fungal species has been classified as cytosolic protein while mammalian or human tyrosinase is characterized as integral membrane protein packed in the melanosomal membrane. Notably, only structural variance is produced by the change in the N-terminal region signal peptides and C-terminal tails while conserved residues in the catalytic pocket of the tyrosinase protein were also observed in different species^7,8^. For instance, low (10–30%) sequence similarity has been reported between the mushroom (mh-Tyr), bacterial (ba-Tyr), and human (hu-Tyr)^61^ while conserved residues have been studied (His^X^ residues) interacting with the catalytic binuclear metal center in mh-Tyr, ba-Tyr, hu-Tyr, and plant tyrosinase (pl-Tyr)^62^. In this context, both the sequence and homology model of human tyrosinase protein were aligned on the mh-Tyr to calculate the similarities in the catalytic pocket (Figs. S1–S3). The sequence alignment results revealed that several residues interacting with the co-crystallized tropolone inhibitor in the 3D crystal structure of tyrosinase from *Agaricus bisporus* mushroom are not conserved in human-Tyrosinase (Fig. S1), except Cu-coordinating histidines as reported earlier^63^. Furthermore, the alignment of 3D structures showed relatively similar conformation for the catalytic pocket in both the mh-Tyr and hu-Tyr proteins (Fig. S2–S3). Therefore, the crystal structure of mh-Tyr was considered as the reference model for the in silico analysis to determine the interaction of selected flavonoids in the catalytic pocket of mh-Tyr using extra precision (XP) docking analysis.

**Figure 2:**
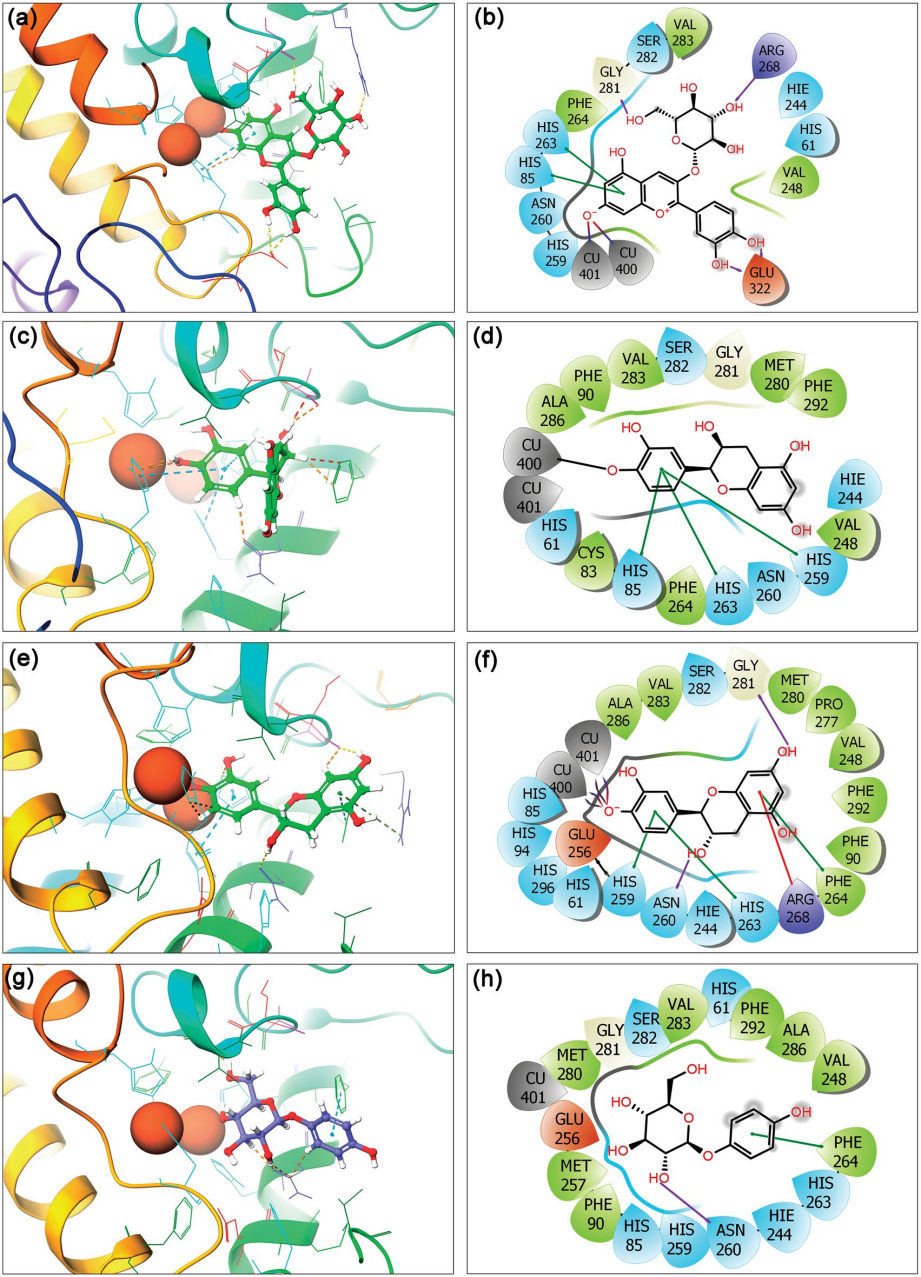
3D and 2D interaction poses for the mh-Tyr protein docked with (**a**, **b**) cyanidin-3-*O*-glucoside (C3G), (**c**, **d**) (−)-epicatechin (EC), (**e**, **f**) (+)-catechin (CH), and (**g**, **h**) arbutin (ARB inhibitor) as positive control. In 2D interaction maps, hydrogen bond (pink arrows), π–π (green lines), π–cation (red lines), hydrophobic (green), polar (blue), negative (red), positive (violet), glycine (grey), metal coordination bond (black line), and salt bridge (red-violet line) interactions are depicted in the respective docked complexes. All the images were generated using free academic Schrödinger-Maestro v12.6 suite^40^; https://www.schrodinger.com/freemaestro.

Initially, the co-crystallized ligand, i.e., tropolone inhibitor as reference ligand, was re-docked in the crystal structure of the mh-Tyr protein to validate the docking protocol. The collected results showed occupancy of tropolone inhibitor in the same pocket with the highest docking energy (− 2.12 kcal/mol) and a slight conformational deviation (1.03 Å) on superimposition over the native conformation in the crystal structure (Fig. S4). Additionally, re-docked reference inhibitor exhibits substantial interactions with active residues (His^61^, His^85^, Phe^90^, His^259^, Asn^260^, His^263^, Phe^264^, Met^280^, Gly^281^, Ser^282^, Val^283^, Ala^286^, and Phe^292^) and binuclear copper ions (CuA^400^ and CuB^401^) via one metal coordination bond (black line), and two salt bridge (red-violet line) formation in the catalytic pocket of mh-Tyr protein against co-crystallized reference ligand (Fig. S5). These results support the considered docking grid and other parameters as ideal for the analysis of selected flavonoids with mh-Tyr. Following, the XP docking of selected flavonoids yields the highest binding affinities between − 9.346 to − 5.301 kcal/mol against the ARB inhibitor (− 5.795 kcal/mol) with mh-Tyr (Table S1, Fig. 2). Thus, the best-docked poses of mh-Tyr with respective compounds at highest negative docking scores were selected for further intermolecular interaction analysis. As depicted in Fig. 2, all the functional groups on A, B, and C-ring of three flavonoids, viz. C3G, EC, and CH, showed differential interactions with the catalytic center of mh-Tyr containing binuclear copper ions (CuA^400^ and CuB^401^) by comparison to the ARB inhibitor. Herein, mh-Tyr-C3G docked complex was noted for the highest docking score of −9.346 kcal/mol and exhibited four hydrogens (H)-bonds at Gly^281^ (C=O⋯H, OH of Glycosyl-ring in C3G: 2.03 Å), Arg^268^ (N−H⋯O, OH of Glycosyl-ring in C3G: 2.06 Å), and Glu^322^ (2; C=O⋯H, OH of B-ring in C3G:1.97 Å and C=O⋯H, OH of B-ring in C3G: 2.20 Å) residues, and interactions with the binuclear copper ions (Cu^400^ and Cu^401^) via salt bridge formation at deprotonated hydroxyl group in the A-ring of C3G. Moreover, hydrophobic (Val^248^, Phe^264^, and Val^283^), polar (His^61^, His^85^, Hie^244^: histidine neutral ε-protonated, His^259^, Asn^260^, His^263^, and Ser^282^), positive (Arg^268^), negative (Glu^322^), glycine (Gly^281^), and π-π (formation via A-ring in C3G with His^85^ and His^263^ residues) intermolecular contacts were also noted in the mh-Tyr-C3G docked complex (Fig. 2a,b). Likewise, molecular docking of EC with the mh-Tyr revealed -6.595 kcal/mol docking energy, contributed by metal coordination bond (Cu^400^) formation at deprotonated hydroxyl group in B-ring of EC along with other intermolecular interactions, including hydrophobic (Phe^90^, Cys^83^, Val^248^, Phe^264^, Met^280^, Val^283^, Ala^286^, and Phe^292^), polar (His^61^, His^85^, His^244^, His^259^, Asn^260^, His^263^, and Ser^282^), glycine (Gly^281^), and π-π bond formation via B-ring in EC (His^85^, His^259^, and His^263^) interactions (Fig. 2c,d). Similarly, the mh-Tyr-CH docked complex was marked for − 5.301 kcal/mol and formed two hydrogen bonds with Asn^260^ (C=O⋯H, OH of C-ring in CH: 2.02 Å) and Gly^281^ (C=O⋯H, OH of A-ring in CH: 2.02 Å) residues. Additionally, salt bridge (Cu^400^ and Cu^401^), metal coordination bond (Cu^400^ and Cu^401^), hydrophobic (Phe^90^, Val^248^, Phe^264^, Pro^277^, Met^280^, Val^283^, Ala^286^, and Phe^292^), polar (His^61^, His^85^, His^94^, His^244^, His^259^, Asn^260^, His^263^, Ser^282^, and His^296^), positive (Arg^268^), negative (Glu^256^), and Glycine (Gly^281^), π–π bond formation via B-ring (His^259^ and His^263^) and A-ring (Phe^264^), and π-cation bond formation via A-ring (Arg^268^) contacts were also recorded in the mh-Tyr-CH docked complex (Fig. 2e,f). However, molecular docking of ARB inhibitor in the active pocket of the mh-Tyr showed a relatively less negative docking score (− 5.795 kcal/mol) and contributed by single H-bond at Asn^260^ (C=O⋯H, OH of Glycosyl-ring in ARB: 1.73 Å), hydrophobic (Phe^90^, Val^248^, Met^257^, Phe^264^, Met^280^, Val^283^, Ala^286^, and Phe^292^), polar (His^61^, His^85^, Hie^244^: histidine neutral ε-protonated, His^259^, Asn^260^, His^263^, and Ser^282^), negative (Glu^256^), glycine (Gly^281^), and π-π bond at phenol-ring of ARB (Phe^264^) interactions (Fig. 2g,h). Of note, all the interacting residues with the docked compounds were the same as in the mh-Tyr crystal structure with tropolone inhibitor^37^.

Importantly, the deprotonation of the selected flavonoids, i.e., C3G, EC, and CH, was observed in the docked poses, suggested that the docked ligands bind to the catalytic pocket of the mh-Tyr as phenolate and presumed to follow a binding mechanism as reported earlier for the mh-Tyr substrate^64,65^. Thus, the released proton is assumed to return in the catalytic pocket of the mh-Tyr to produce water and the quinone product^65^. Moreover, geometrically, the positioning of B-ring in the tyrosinase inhibitors approximately orthogonal to the plane connecting the coupling ions with ~ 90° has been characterized as an ideal orientation required by Quintox mechanism^65^, which results in the inactivation of tyrosinase^66^. Remarkably, the B-ring in EC and CH was noted to occupy similar plane and molecular contact formations with the catalytic residues of the mh-Tyr against C3G and ARB inhibitor; and hence, EC and CH were elucidated to possess favorable geometric orientation for the cresolase-like pathway to exhibit tyrosinase inhibition (Fig. 2). Based on these observations, EC and CH were predicted to exhibit the inactivation of tyrosinase enzyme by competing with or delaying the oxidation of substrate as reported earlier for Epicatechin gallate (ECG)^66^. Collectively, based on the docking energy and intermolecular interactions analysis of docked poses, these results suggested that the selected flavonoids, i.e., C3G, EC, and CH, could interact with both metal ions and essential residues in the catalytic pocket of the mh-Tyr in reference to ARB inhibitor.

### Molecular dynamics simulation analysis

**Figure 3:**
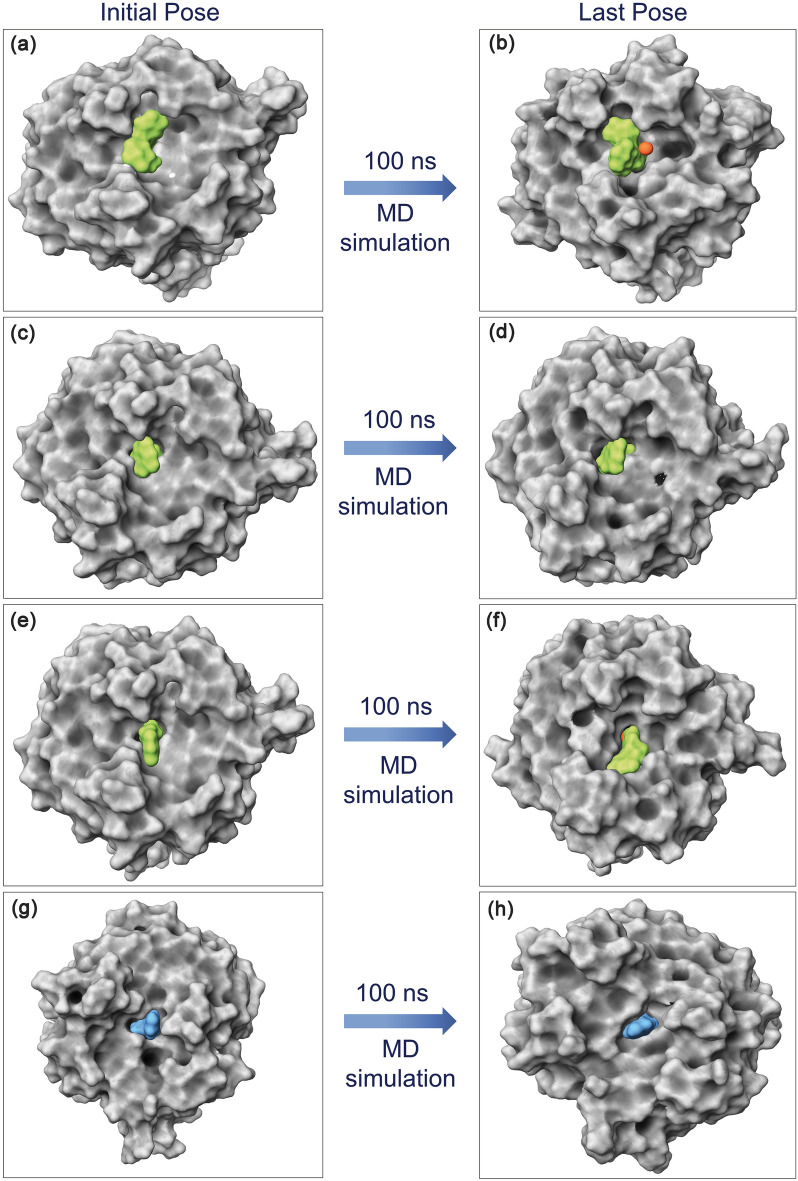
3D surface poses of the docked mh-Tyr as receptor with selected compounds, i.e., (**a**, **b**) C3G, (**c**, **d**) EC, (**e**, **f**) CH, and (**g**, **h**) ARB inhibitor, representing the conformation changes through 100 ns MD simulation. Herein, 3D images were generated using free academic Schrödinger-Maestro v12.6 suite^40^; https://www.schrodinger.com/freemaestro.

Physics-based molecular dynamics (MD) simulation in principle allowed the demonstration of optimized protein–ligand binding and unbinding process^67,68^ and have been associated with improved drug development approaches^69–71^. Moreover, MD simulation is solely used in drug discovery to predict the conformation changes and intermolecular interaction profiling at the molecular level as a function of simulation interval^72–74^. Thus, analysis of docked complex stability and induced conformational changes in the local structures of the docked species using the MD simulation can provide substantial insights into the understanding of protein inhibition. Initially, MD simulation performed for the mh-Tyr reference complex showed acceptable (< 3 Å, with expectation for higher RMSF in the loop region > 4 Å) root-mean-square deviation (RMSD) and root-mean-square fluctuation (RMSF) values for both the protein and ligand as a function of 100 ns interval, (Figs. S6–S8), indicates the substantial stability of the re-docked mh-Tyr-reference inhibitor complex. Hence, these observations marked the considered simulation parameters as ideal MD simulation setup to evaluate the stability of the mh-Tyr-flavonoids complexes. Following, MD simulation of all the docked flavonoids with mh-Tyr also exhibits considerable global minimum within 20 ns interval while ligands retained in the catalytic pocket of the mh-Tyr during the 100 ns interval by comparison to the positive inhibitor (Fig. 3). Hence, each generated MD trajectory (for mh-Tyr-flavonoids and mh-Tyr-positive inhibitor complexes only) was further analyzed for the (i) last MD trajectory pose (a single protein–ligand complex structure) molecular contacts formation after attaining global minima for the docked complex, (ii) statistical analysis of the complete MD trajectory in terms of root mean square deviation (RMSD) and root mean square fluctuation (RMSF), and (iii) complete intermolecular interactions by protein–ligand contact mapping method in the simulation interaction diagram tool of the free academic version of Desmond suite.

#### Last pose molecular contact profiling

**Figure 4:**
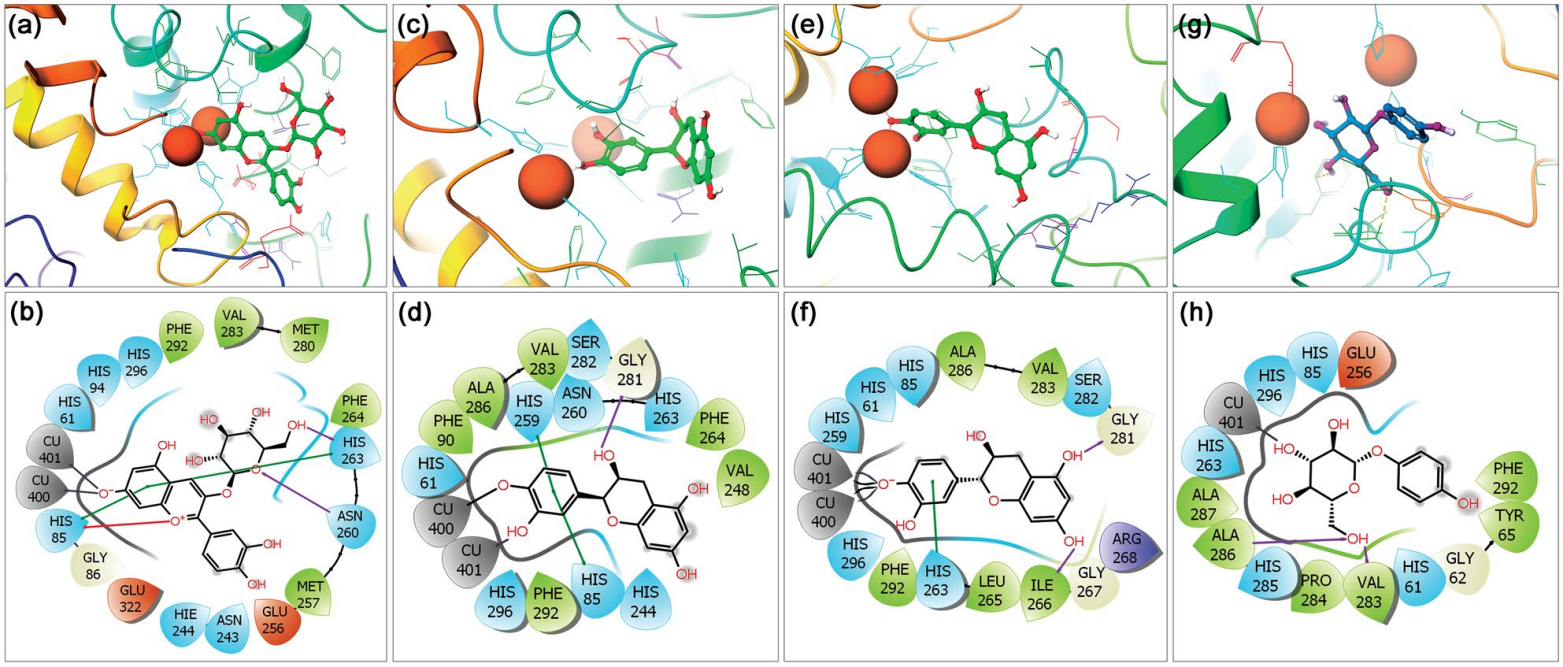
3D and 2D interaction analysis in the extracted last poses for the mh-Tyr docked with (**a**, **b**) C3G, (**c**, **d**) EC, (**e**, **f**) CH, and (**g**, **h**) ARB inhibitor. In 2D interaction maps, hydrogen bond (pink arrows), π–π (green lines), π–cation (red lines), hydrophobic (green), polar (blue), negative (red), positive (violet), glycine (grey), metal coordination bond (black line), and salt bridge (red-violet line) interactions are depicted in the respective extracted snapshots. All the 3D and 2D images were generated by free academic Schrödinger-Maestro v12.6 suite^40^; https://www.schrodinger.com/freemaestro.

First, to determine the stability of docked ligands in the catalytic pocket of the mh-Tyr enzyme, the last poses were extracted from respective 100 ns MD simulation trajectories and analyzed for the displacement of docked ligands against the respective initial docked poses. Figure 3 shows no significant alteration in the docked compounds conformation after 100 ns MD simulation in reference to initial poses, suggesting that docked ligands maintained the strong interactions with essential residues in the catalytic pocket during MD simulation interval and established the formation of stable complexes. Therefore, these last poses were further computed for the intermolecular interactions between the atoms of the selected compounds and active residues in the binding pocket of the mh-Tyr protein (Table S2, Fig. 4).

Notably, at least two hydrogen bond formations were noted in all the complexes, except one H-bond was observed in the mh-Tyr-EC and mh-Tyr-C3G complexes, while π–π or *π–cation interactions were also noted with the active residues in the mh-Tyr-C3G complex (Fig. 4). Additionally, each docked flavonoid demonstrated interactions with the binuclear copper via metal coordination bond formation against positive control, i.e., ARB inhibitor, which formed only a single metal coordination bond with one copper ion (Cu^401^) present in the catalytic pocket of the protein (Fig. 4). These molecular contacts profiles in each last pose were the same as in the docked complexes (Table S1, Fig. 2), suggesting the significant interactions of selected bioactive compounds, i.e., C3G, EC, and CH, with the active residues of the mh-Tyr. Of note, MD simulation using Desmond algorithm has been reported significantly to capture the small molecule distinguishing and attaching to a receptor using long and unbiased MD simulation, which was typically identical to the experimentally defined crystal structure^75^. Hence, these collected results established the substantial stability of the docked flavonoids with mh-Tyr and to function as an alternative substrate in presence of a specific substrate to reduce or inhibit the catalytic activity of the mh-Tyr enzyme, as predicted from docked poses (Fig. 2).

#### Root-mean square deviation and fluctuation analysis

**Figure 5:**
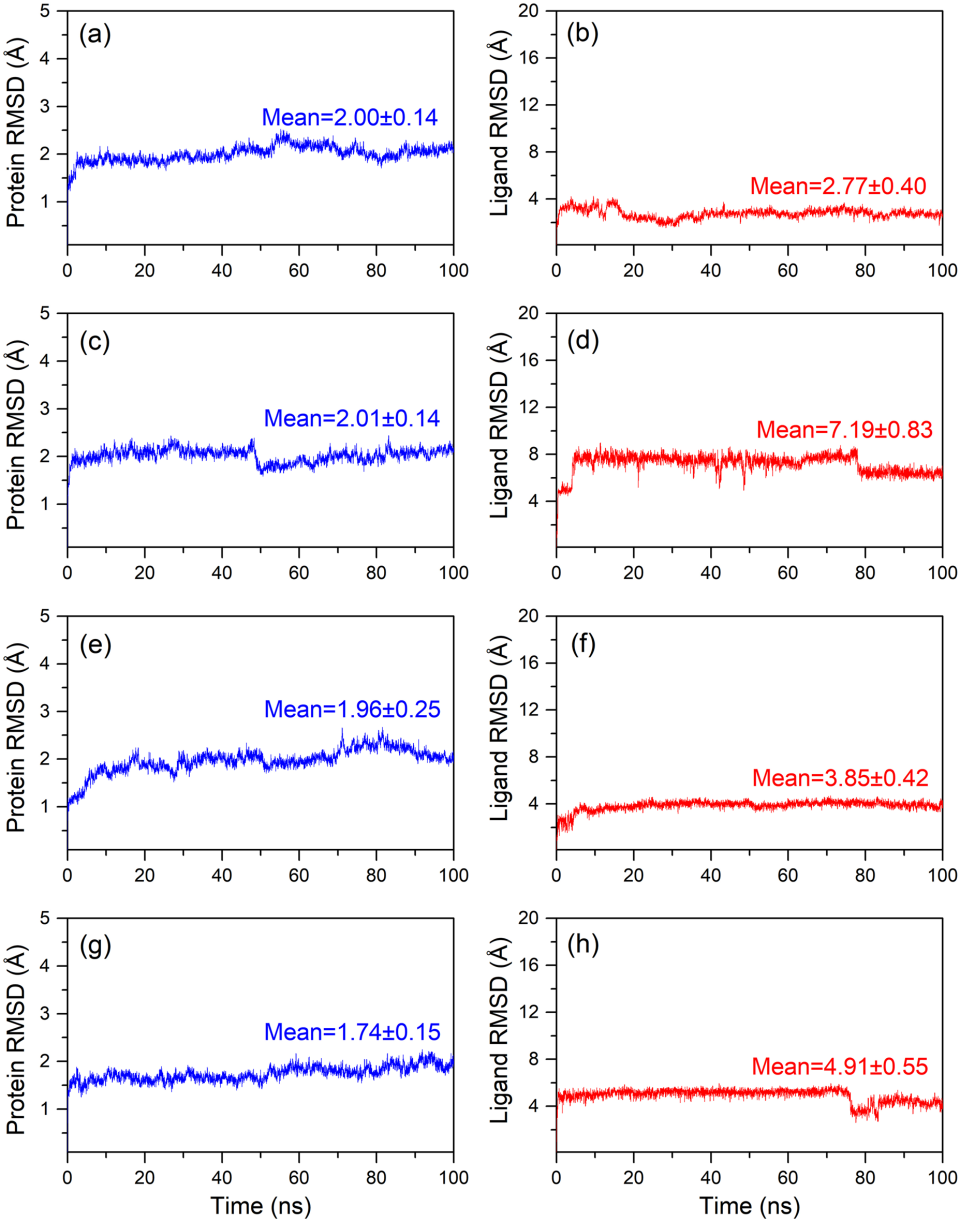
RMSD values plotted for the docked mh-Tyr protein and mh-Tyr fit ligands extracted from simulated complexes, i.e., (**a**, **b**) mh-Tyr-C3G, (**c**, **d**) mh-Tyr-EC, (**e**, **f**) mh-Tyr-CH, and (**g**, **h**) mh-Tyr-ARB inhibitor, with respect to 100 ns simulation interval.

Root-mean-square deviation (RMSD) is the most frequently used measure for structure comparison in structural biology, including monitoring the structural changes or characterizing the quality of the structure in protein folding and dynamics^76,77^. Typically, RMSD is often analyzed for backbone atoms by reporting its arithmetic mean in computer simulations^78^. Likewise, root-mean-square deviation (RMSF) is widely used on the ensemble of structures or MD trajectory to extract the fluctuations of an atomic position approximately it’s average value^79^. Therefore, to monitor the structural variations and quality of each docked receptor-ligand complex, RMSD and RMSF values for the (α)alpha-carbon atoms of the protein were calculated in reference to the first pose of the MD simulation and analyzed by comparison to the respective values of the α-carbon atoms in the apo-mh-Tyr structure (Figs. 5, S9–S12). Here, a slight increase (< 0.1 Å) in the RMSD values for the docked mh-Tyr against apo-mh-Tyr in the initial phase signifies the structural adjustments in the system due to ligand binding in the catalytic pocket during the simulation process. However, all the protein structures in each docked complex with flavonoids later demonstrated no deviations and were noted for acceptable RMSD values (< 2.01 Å) against the mh-Tyr-ARB inhibitor complex (< 1.74 Å) and apo-mh-Tyr (< 2.57 Å) till the end of 100 ns MD simulation (Figs. 5, S9). Overall, the RMSD plots for the protein indicated that docking of the selected compounds in the active pocket of mh-Tyr have induced rigidity and formed a stable conformation against the apo-mh-Tyr structure as predicted in the docked poses and respective extracted last poses from the MD simulation trajectories (Figs. 2, 4). These observations were also supported by the reduced RMSF values (< 3 Å) for the backbone in the docked protein, except occasional high RMSF values (< 3.2 Å) were noted for the residues in the adjutant regions or directly interacting with the docked ligands, against apo-mh-Tyr structure (< 5 Å) (Figs. S10, S11). For instance, RMSF noted for the mh-Tyr-C3G complex exhibited reduced RMSF in the residues directly interacting with the ligand (in loop region) while higher RMSF was noted in the adjusted residues (in loop region) for the EC, CH and ARB docked complexes with mh-Tyr (Fig. S11). Additionally, substantial fluctuations were noted in the N-terminal, loops, and outer regions of the apo-mh-Tyr (Fig. S10), indicating the greater flexibility of protein in absence of ligand in the active pocket during MD simulation. Baweja et al. suggested that the residues situated in the inner regions of protein exhibit low RMSF values followed by high RMSF values in the loop regions and residues located on the protein surface^80^. Thus, observed variations in the docked protein structures were considered acceptable and predicted to contribute by the binding or unbinding of respective ligands during the MD simulation interval.

Furthermore, protein fit ligands were also analyzed for the RMSD values in reference to the first poses during the initial interval of the 100 ns MD simulation (Fig. 5). Herein, only mh-Tyr fit C3G (2.77 Å RMSD) exhibited ideal average deviations against EC (7.19 Å RMSD), CH (3.85 Å RMSD), and ARB inhibitor (4.91 Å RMSD) (Fig. 5). Analysis of the simulation trajectory in the form of MD simulation movie revealed substantial displacement in A and C-ring (non-metallic interactions with mh-Tyr) against B-ring (displaying metal-coordination bonds with mh-Tyr) of EC and CH that contributed to the rapid increase in the RMSD during the initial interval of the MD simulation (MD Movie S1–S3, Fig. 5). Similar considerable displacement in the phenolic ring (non-metallic interactions) against glucopyranoside ring (metallic interactions) in the ARB inhibitor was noted during the initial phase and between 75 and 80 ns interval of MD simulation that added  a deviation in RMSD to the mh-Tyr-fit ARB inhibitor as a function of 100 ns interval (MD Movie S4, Fig. 5). Of note, all the docked flavonoids maintained the state of equilibrium (variation < 1 Å) along the trajectory and interactions with the binuclear metal ions as a function of time (Fig. 5). Additionally, the calculated protein fit ligand RMSD values were also favored by acceptable respective RMSF values (< 2 Å), except occasional high RMSF values (< 4.2 Å) in the atoms of the C3G and EC were observed against positive control (< 2 Å) (Fig. S12). These atomic fluctuations in the docked flavonoids are predicted to be induced by chelation with binuclear copper ions and active residues in the binding pocket of the mh-Tyr as noted in the respective extracted last poses and MD movie analysis of the simulation trajectories (MD Movies S1–S4, Fig. 4). Collectively, RMSD and RMSF values signify the global minima required for the durable stability of each docked complex of mh-Tyr with selected flavonoids, i.e., C3G, EC, and CH, against positive control, viz. ARB inhibitor, where mh-Tyr-C3G complex was concluded for higher stability and interactions with mh-Tyr against other docked flavonoids and positive inhibitor.

#### Protein–ligand contact mapping

**Figure 6:**
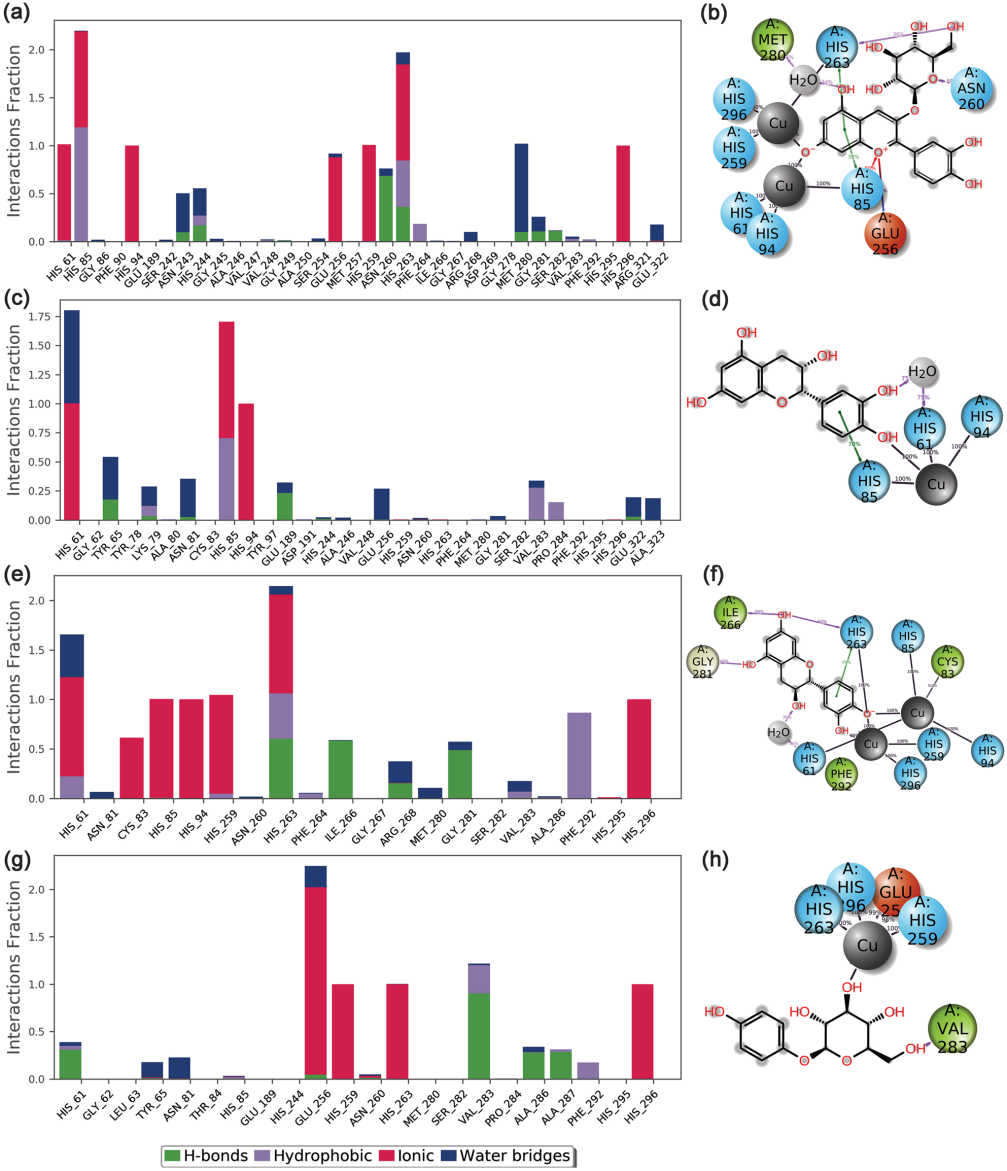
Protein–Ligand contact mapping for the mh-Tyr protein docked with selected compounds, i.e., (**a**, **b**) C3G, (**c**, **d**) EC, (**e**, **f**) CH, and (**g**, **h**) ARB inhibitor, extracted as a function of 100 ns MD simulation interval. Herein, 2D interaction maps exhibit 30% of the total interaction that occurred between the atoms and residues in the respective docked complexes. These images were rendered using the SID module in the free academic version of Desmond v5.6^49^; https://www.deshawresearch.com/resources_desmond.html.

To further comprehend the total intermolecular interactions between the docked mh-Tyr and selected compounds, protein–ligand contact maps were plotted for each docked complex from the respective 100 ns MD simulation trajectory. Typically, H-bond formation in the receptor-ligand complex has been reported to understand the structural stability and interactions with the docked ligands^81,82^. Besides, hydrophobic, ionic, polar, and water bridge-hydrogen-bonded interactions have been well established as key factors that contributed to the stability of the docked complex during the MD simulation. Hence, all these interactions were extracted for each docked complex from the respective 100 ns MD simulation trajectory under default parameters in the free academic version of the Desmond module (Fig. 6).

Remarkably, all the docked flavonoids with mh-Tyr protein showed substantial molecular contact formation (100% percent or more than 100% interaction fraction of the total simulation interval) with residues coordinating with the binuclear copper ions, i.e., His^61^, His^85^, His^94^, His^259^, and His^263^, and other essential residues (Phe^90^ and Phe^292^) in the binding pocket (Fig. 6). Herein, the mh-Tyr docked with C3G showed 100% interaction fraction of the total simulation interval with His^61^, His^94^, Glu^256^, His^259^, and His^296^ residues as well as multiple intermolecular interactions (more than 100% interaction fraction of the total simulation interval), such as hydrogen bonding, hydrophobic, and water bridge formation at His^85^, Asn^243^, His^244^, Asn^260^, His^263^, and Met^280^ residues. Likewise, the mh-Tyr-EC complex showed 100% interaction fraction at His^91^ and substantial multiple molecular contacts formation at His^61^, Tyr^65^, His^85^, and Glu^189^ residues during the 100 ns MD simulation interval. Whereas in mh-Tyr-CH complex, docked ligand exhibited 100% ionic interaction fractions (His^85^ and His^95^ residues) and multiple intermolecular interaction fractions (His^61^, Cys^83^, His^259^, His^263^, Ile^266^, Arg^268^, Gly^281^, Val^283^, Phe^292^, and His^296^ residues) in the active pocket of mh-Tyr. Whereas positive control complex, i.e., mh-Tyr- ARB inhibitor, was noted for only ionic interaction (100% interaction fraction of the total simulation interval) at His^259^, His^263^, and His^296^ residues as well as multiple interaction fractions, including hydrophobic, hydrogen bond, and water bridge formations at Glu^256^, Val^283^, Ala^286^, and Ala^287^ residues. Furthermore, interaction fraction at 30% of the total simulation was also extracted for each docked complex from the respective MD trajectories. Figure 6 revealed the interaction of C3G via A-ring, EC and CH showed interaction through B-ring, and ARB inhibitor exhibits substantial contacts by glucoside group with the catalytic center of mh-Tyr. Hence, during MD simulation, the selected flavonoids (C3G, EC, and CH) against positive control, i.e., ARB inhibitor, were established for metal-coordination bond formation with binuclear copper ions present in the catalytic pocket of mh-Tyr, which are essentially required to perform the catalysis of phenols into *o*-quinones^9,16^. Furthermore, number of intermolecular contacts formation and their density (darker shade of orange indicates more than one contact on that frame with the residues) for the respective docked flavonoid and positive control complexes were also studied from the 100 ns MD simulation trajectories (Fig. S13). Based on these observations, the docked compounds can be arranged in the order of substantial interactions with the active residues of the mh-Tyr during the 100 ns MD simulation interval, viz. C3G > CH > EC > ARB inhibitor. Therefore, screened flavonoids were assumed to function as potent alternative substrates of the mh-Tyr protein by comparison to positive control. i.e., ARB inhibitor.

#### Principal component analysis

**Figure 7:**
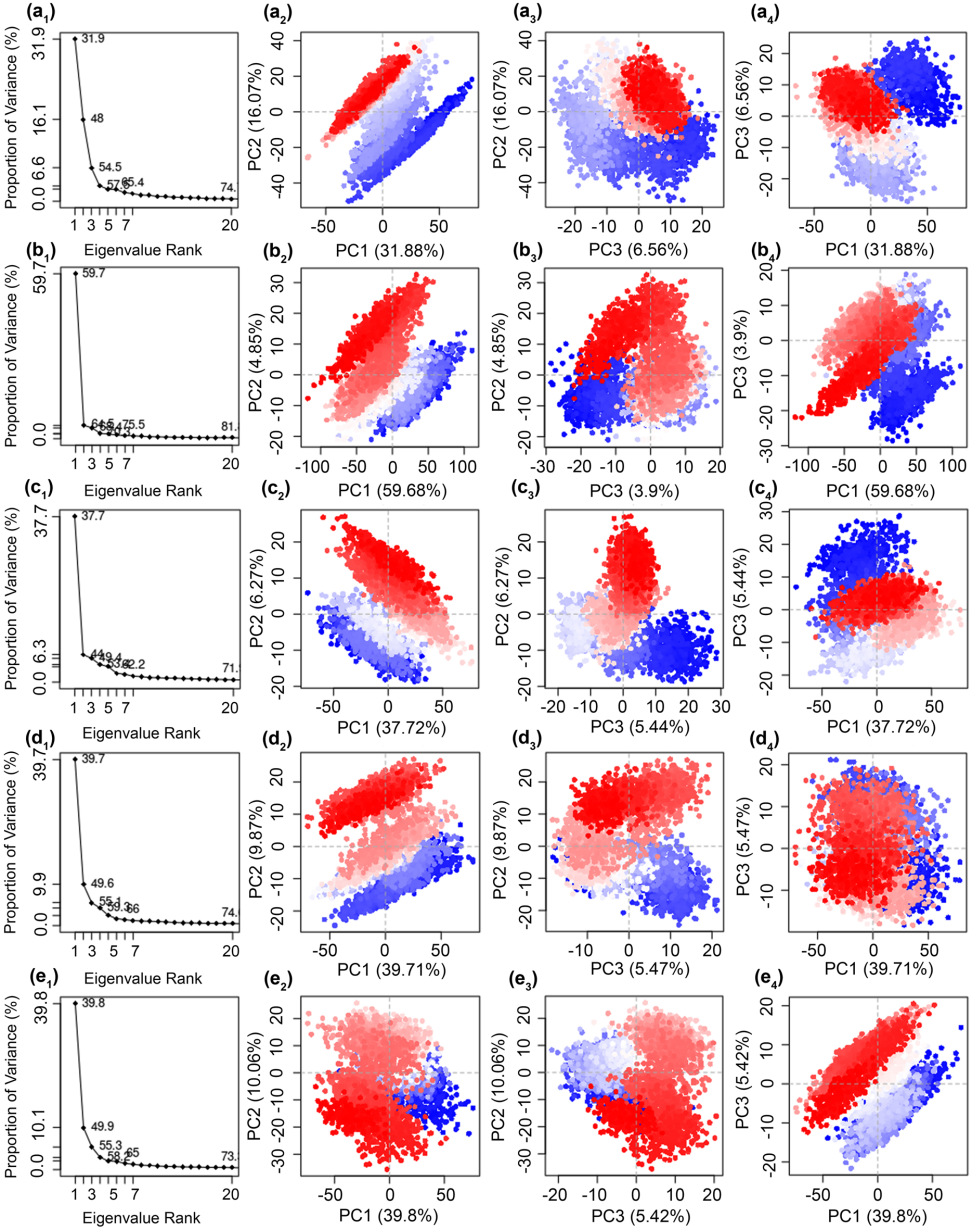
Principal component analysis of the mh-Tyr docked complexes with (**a**) C3G, (**b**) EC, (**c**) CH, and (**d**) ARB inhibitor against the (**e**) apo-mh-Tyr protein. The instantaneous conformations of mh-Tyr protein are colored from blue to red through white color in order of time (0–100 ns) in the respective scatter plots, which signify the periodic jumps at different intervals of the 100 ns MD simulation. Images were generated using default parameters in Bio3d package (Released version 2.4–1; http://thegrantlab.org/bio3d/)^51^ under R environment (R version 4.0.4; http://mirror.fcaglp.unlp.edu.ar/CRAN/)^52^.

Protein activity is modulated by the collective fluctuations in the atoms of the residues and by achieving various conformations. To collect the essential motions in the mh-Tyr structure before and after docking with the selected compounds using respective MD simulation trajectories, essential dynamics via principal component analysis was performed on the collected 10,000 frames from MD simulation trajectory by the projection of principal components (orthogonal eigenvectors) under default parameters in the Bio3D package. Herein, a total of 20 eigenvalues were collected corresponding to each eigenvector to understand the dynamic behavior of the protein (Fig. 7). Among the docked poses, mh-Tyr-C3G (~ 65.4%), mh-Tyr-EC (~ 75.5%), mh-Tyr-CH (~ 62.2%), and mh-Tyr-ABR (~ 59.66%) exhibited a steep drop in the Eigen fraction corresponds to the early five eigenvalues by comparison to apo-mh-Tyr structure (58.65%). Of note, mh-Tyr-EC and mh-Tyr-CH complexes showed a rapid reduction in the proportion of variance in the protein within the early three eigenvalues, indicating a rapid reduction in protein flexibility by the docked EC and CH by comparison to C3G and ARB inhibitor. Also, a consecutive elbow point at the 5^th^ eigenvalue and no further substantial changes till the 20th eigenvalue supported the convergence or equilibrium state for the mh-Tyr structure (Fig. 7). Collectively, these observations suggested that binding of EC and CH causes a substantial reduction in protein essential motions against C3G and ARB inhibitor during the initial interval of MD simulation which eventually equilibrated to a stable conformation as a function of 100 ns interval. Notably, a similar prediction was extracted from the trajectory analysis of respective complexes (Fig. 5).

Furthermore, the first three eigenvectors were collected from each MD simulation trajectory and plotted to demonstrate the residual displacement in the different conformations of the protein structure, where a gradient color change (from blue to white to red) specifies that there are regular leaps among the various conformation of protein structure throughout the trajectory (Fig. 7). Of note, projection of the first two PCs (PC1 and PC2), which covered maximum variations, showed a considerable compact cluster distribution (centered between − 50 to + 50 plane) for the residual motion in the mh-Tyr structure docked with all the ligands during 100 ns simulation, except in mh-Tyr-EC complex (centered between − 100 to + 100 plane), by comparison to apo-mh-Tyr (centered between − 50 to + 50 plane) (Fig. 7). However, each system was observed with uniform distribution in more compact or similar planes for the projected PC2 vs PC3 (centered between − 10 to + 30 plane) and PC3 vs PC1 (centered between − 50 to + 100 plane), indicating the state of equilibrium for the mh-Tyr docked conformations by comparison to apo-mh-Tyr during the simulation. Recently, intermolecular contact formed by brazilein, identified as an oxidized form of brazilin (neoflavonoid), via copper chelation along with hydrophobic and hydrogen bonding in the catalytic core of tyrosinase was established to induce structural variations in the secondary structure of the protein^83^. Conclusively, the subsequent decrease in correlated and compact motions in mh-Tyr structure in respective docked complexes against apo-protein demonstrated the substantial stability of the respective docked complexes during MD simulation.

### Net binding free energy analysis

**Figure 8:**
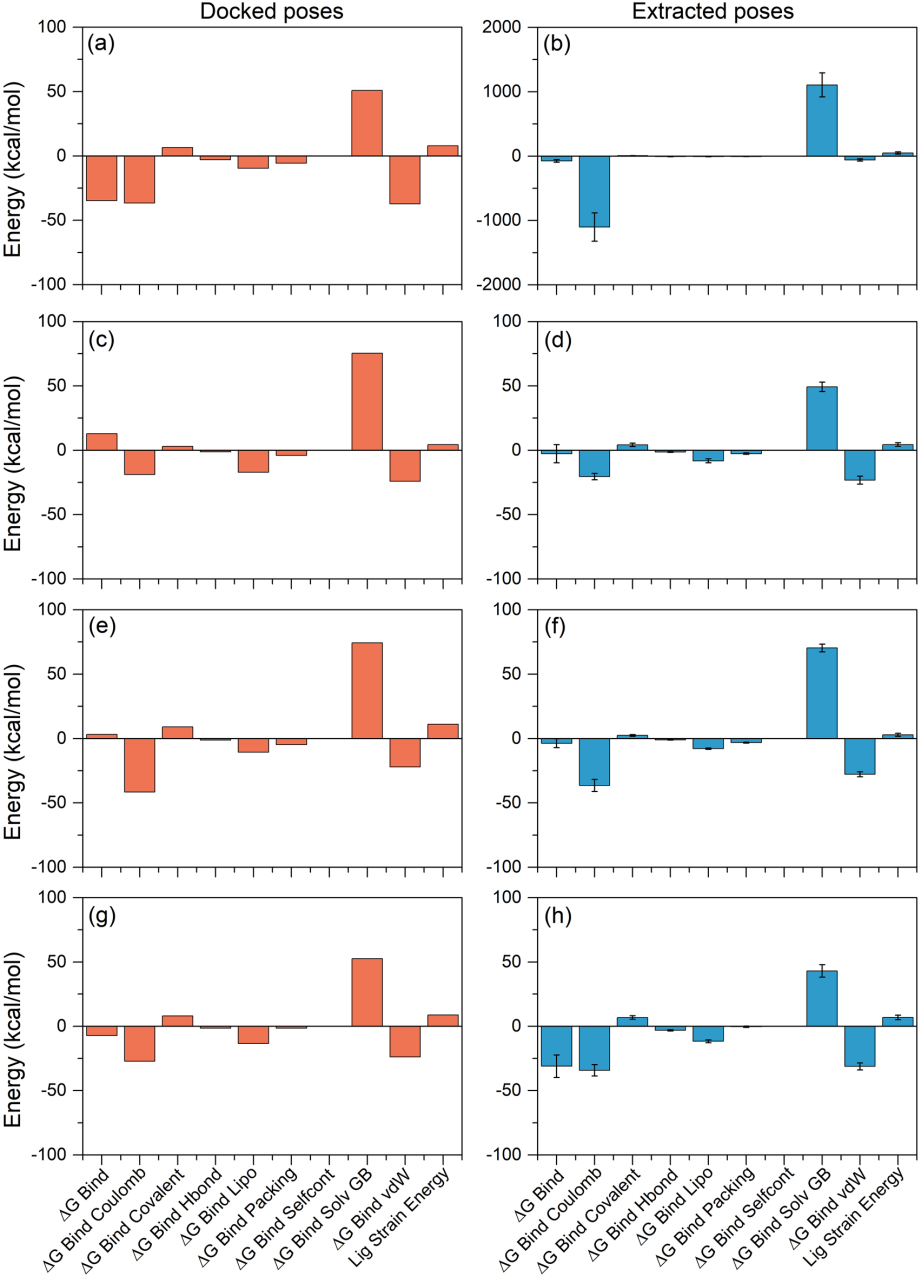
Net MM/GBSA binding free energy and energy dissociation components (kcal/mol) calculated for the docked poses (orange color) and MD simulation extracted poses (Blue color) with standard deviation values for the mh-Tyr docked complexes with selected bioactive compounds, i.e. (**a**, **b**) C3G, (**c**, **d**) EC, (**e**, **f**) CH, and (**g**, **h**) ARB inhibitor.

Molecular mechanics generalized Born surface area (MM/GBSA) approach was used to calculate the total binding free energy and energy dissociation components that added to the stability of docked mh-Tyr complexes with selected compounds. Herein, to demonstrate the difference in the net binding energy before and after MD simulation, the respective docked poses and extracted snapshots (from the last 10 ns interval of respective MD simulation trajectories) were subjected to comparative free binding energy analysis (Table S3). As shown in Fig. 8, the highest negative binding free energy was noticed for the mh-Tyr-C3G docked complex (− 34.72 kcal/mol) by comparison to mh-Tyr-ARB inhibitor complex (− 7.23 kcal/mol) while docked complexes of mh-Tyr-EC (12.84 kcal/mol) and mh-Tyr-CH complex (3.1 kcal/mol) exhibited a net positive binding energy. However, snapshots collected from the last 10 ns MD simulation trajectory of the mh-Tyr-C3G docked complex (− 74.51 ± 20.49 kcal/mol) revealed substantial binding free energy against positive control, i.e., mh-Tyr-ARB inhibitor complex (− 31.09 ± 8.76 kcal/mol). Moreover, the least free binding energy was observed for the extracted poses of mh-Tyr-EC (− 2.67 ± 7.03 kcal/mol) and mh-Tyr-CH (− 3.68 ± 3.47 kcal/mol) from the respective MD simulation trajectories (Fig. 8). Besides, energy dissociation component analysis revealed the contribution of ΔG_*Bind Coulomb*_ (Coulomb energy) and Δ*G*_*Bind vdW*_ (Van der Waals interaction energy) to the stability of the complex while ΔG_*Bind Covalent*_ (Covalent energy) and ΔG_*Bind Solv GB*_ (Generalized Born electrostatic solvation energy) tends to separate the interacting receptor and ligand in both the docked complexes and during MD simulation (Table S3, Fig. 8). Additionally, the role of ΔG_*Bind Hbond*_ (H-bonding correction), ΔG_*Bind Lipo*_ (Lipophilic energy), and ΔG_*Bind Packing*_ (π-π packing correction) were also marked for contribution to the stability of the respective docked complexes while no contribution of ΔG_*Bind Self Cont*_ (Self-contact correction) was observed in each complex (Table S3, Fig. 8).

Also, calculated ligand strain energy revealed the substantial contribution in the mh-Tyr-C3G complex during MD simulation against other docked complexes of the mh-Tyr (Fig. 8). Interestingly, in this study, docked poses of the mh-Tyr-EC and mh-Tyr-CH showed positive binding free energy when interacting with copper ions while endpoint binding free energy exhibits lower negative energy values (Table S3, Fig. 8). Thus, the intermolecular interactions of docked ligands with metal ions in the mh-Tyr were predicted to cause a reduction in the net binding free energy for the mh-Tyr-EC and mh-Tyr-CH complexes using MM/GBSA method. Furthermore, a recent analysis of catechins from green tea with mh-Tyr found that although epigallocatechin gallate (EGCG) showed higher free binding energy but noted for least mh-Tyr inhibition by comparison to catechin due to the lack of the catechol group^66^; this observation advocates the substantial interaction between the catechol group in catechins with the catalytic cavity for the mh-Tyr inhibition. Hence, C3G was marked to form the most stable complex with mh-Tyr; however, lack of interactions from the catechol group, as observed in docked poses and MD analysis, predicted to cause weak or no mh-Tyr inhibition by comparison to other selected flavonoids (EC and CH) due to rapid oxidation in the catalytic pocket of the mh-Tyr protein.

### Mushroom tyrosinase inhibition assay

**Figure 9:**
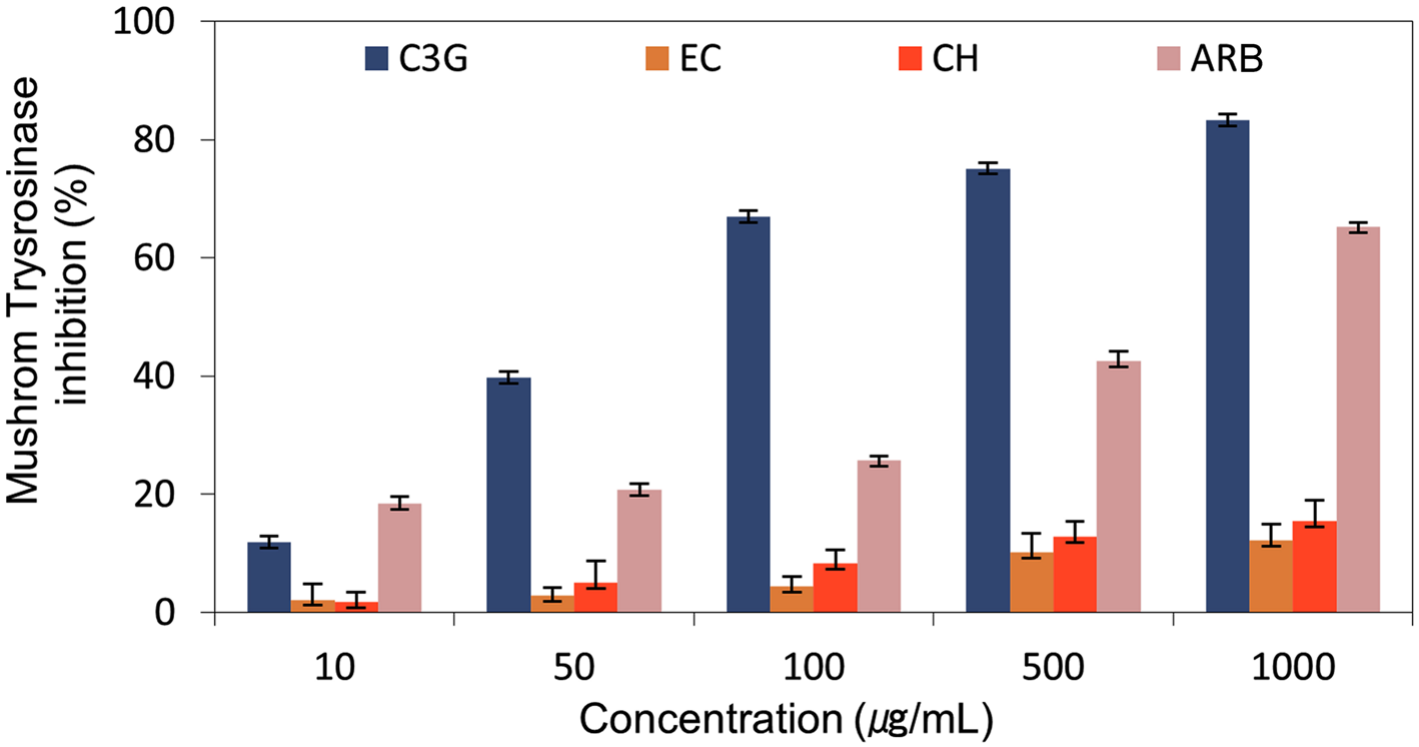
Mushroom tyrosinase (mh-Tyr) inhibition profiling for the selected bioactive compounds, i.e., C3G, EC, and CH, against positive control compound, viz. ARB inhibitor, using spectrophotometry method.

To evaluate the inhibition of the mh-Tyr by the selected flavonoids, i.e., C3G, EC, and CH, against positive control, i.e., ARB inhibitor, two different approaches, including in vitro mh-Tyr inhibition using spectrophotometer method and visual examination of enzyme inhibition by zymography method, were used to monitor the mh-Tyr activity under different concentrations of the respective compounds (Table S4). Figure 9 exhibits results for the inhibition of the mh-Tyr calculated using a spectrophotometer, where a dose-dependent inhibition of the mh-Tyr was exhibited by the selected flavonoids against positive control. Notably, C3G (83.2% at 1000 μg/mL) was measured for highest inhibition by comparison to ARB inhibitor (65.2% at 1000 μg/mL). However, no substantial effect of EC (12.1% at 1000 μg/mL) and CH (15.4% at 1000 μg/mL) was noted in the mh-Tyr inhibition (Table S4, Fig. 9). These results revealed C3G as a potential inhibitor of the mh-Tyr against other bioactive compounds (EC and CH) and positive control (ARB inhibitor).

**Figure 10:**
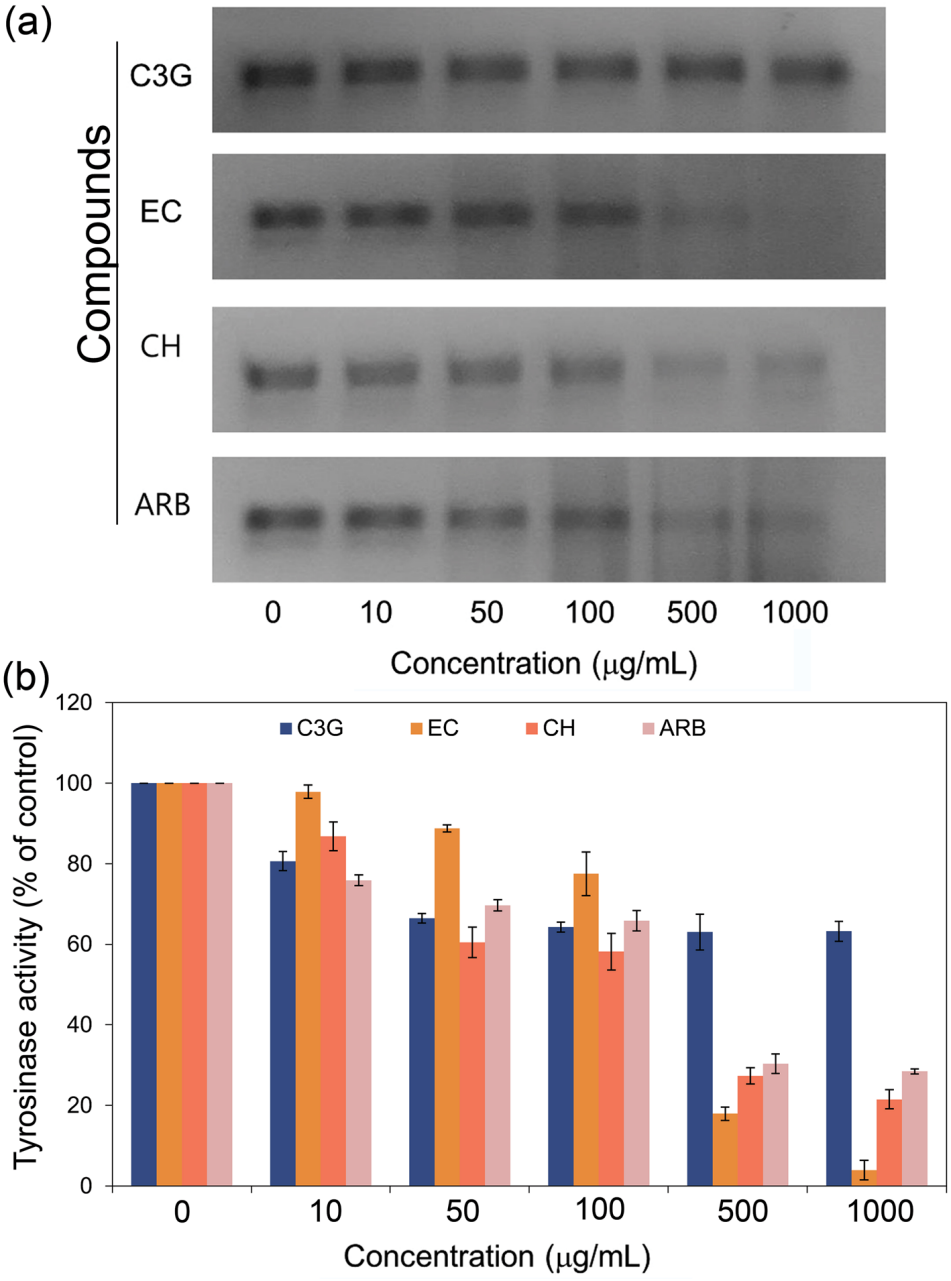
Zymograms analysis for the inhibition of the mh-Tyr enzyme incubated with different concentrations of selected bioactive compounds, i.e., C3G, EC, and CH, and positive control compound, viz. ARB inhibitor. Herein (**a**) zymograms show the dark black to faded black color bands corresponding to the *o*-quinone production by the activity of mh-Tyr and (**b**) measured color intensity of the bands with standard deviations from the triplicate experimental data.

To validate the mh-Tyr inhibition caused by the selected compounds without interference with the absorption spectra, tyrosinase zymogram analysis was conducted on the selected concentrations for the flavonoids and positive control (Table S5, Figs. S14–S17, Fig. 10). Remarkably, no significant inhibition in the mh-Tyr activity was observed after 50 μg/mL incubated with C3G while both EC and CH exhibited a concentration-dependent reduction in the mh-Tyr activity against ARB inhibitor (Fig. 10). Herein, a maximum mh-Tyr activity of 63.2, 3.9, 21.5, and 28.4% were determined at a maximum concentration (1000 μg/mol) for the C3G, EC, CH, and ARB inhibitor, respectively from the respective mh-Tyr zymograms (Table S5, Fig. 10). Of note, these results were in contradiction with the calculated mh-Tyr inhibition using the spectrophotometer method (Fig. 8). Thus, observed results from the spectrophotometer method suggested the interference of flavonoids with the elucidation of mh-Tyr inhibition as reported previously^29^. Hence, based on the visual observations of the zymograms, EC and CH were concluded as potent inhibitors of the mh-Tyr enzyme against ARB inhibitor.

### Cell viability and cell-free tyrosinase inhibition assay

**Figure 11:**
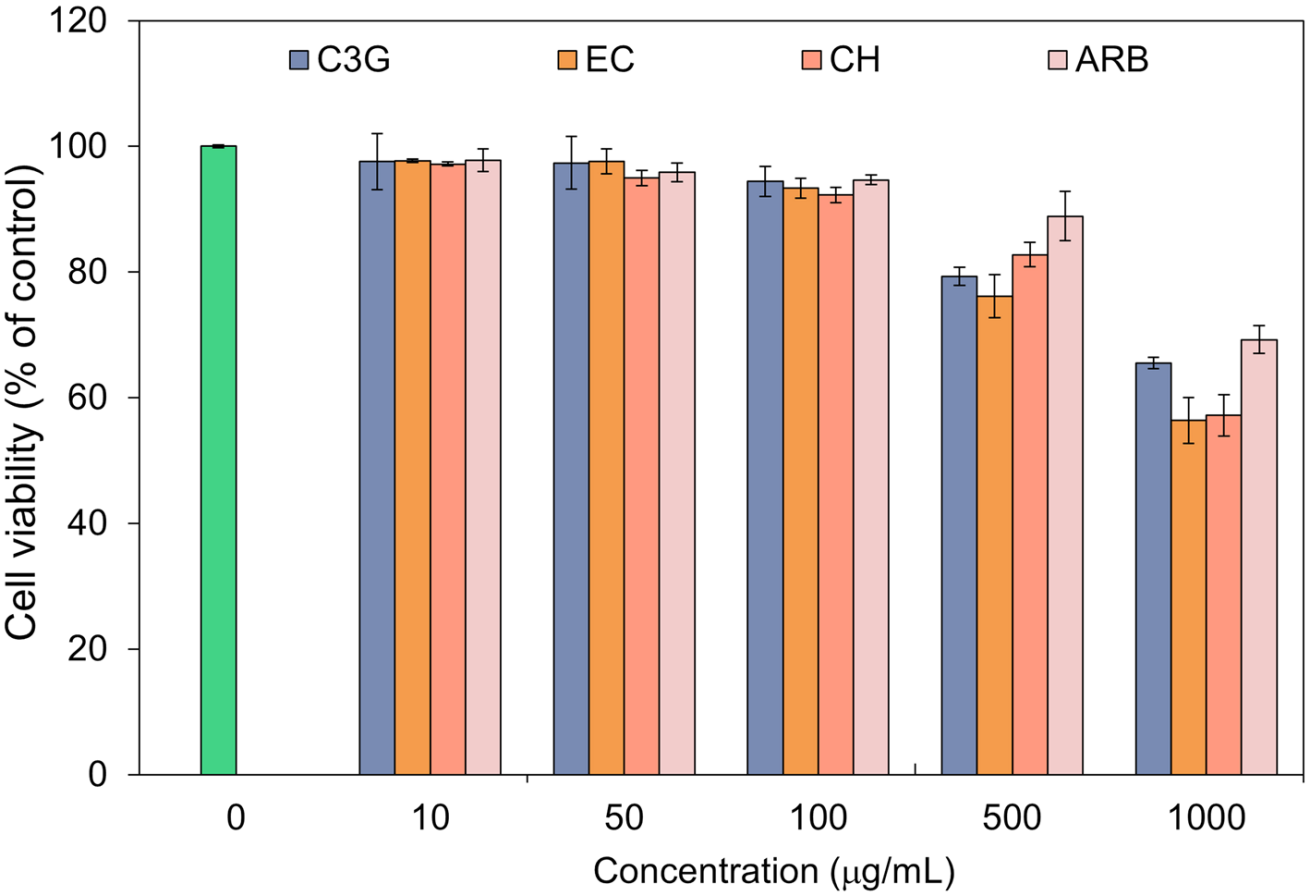
Cell viability profiling for the selected bioactive compounds, i.e., C3G, EC, and CH, and positive control compound, viz. ARB inhibitor, at different concentrations (10–1000 μg/mL) on the murine melanoma B16F10 cell culture by comparison to positive control.

Considering the potential of selected flavonoids as mh-Tyr inhibitors and so as an active ingredient for the formulation against hyperpigmentation, evaluation of these compounds for their cell viability efficacy in mammalian cell lines is required before furthering the experimental analysis. Therefore, murine melanoma B16F10 cell culture was selected to perform the in vitro efficacy assay for the selected flavonoids against positive control (Table S6, Fig. 11). Remarkably, no substantial toxicity (~ 98% viable cells) to the cell was observed at lower concentrations (10–100 μg/mL). A further increment in the concentration of each compound resulted in a substantial reduction in the percentage of viable cells by comparison to control (no treatment) (Table S6, Fig. 11). Hence, a moderate concentration (100 μg/mL), which showed no substantial reduction in viable cells, was considered for each selected compound for further experimental analysis.

**Figure 12:**
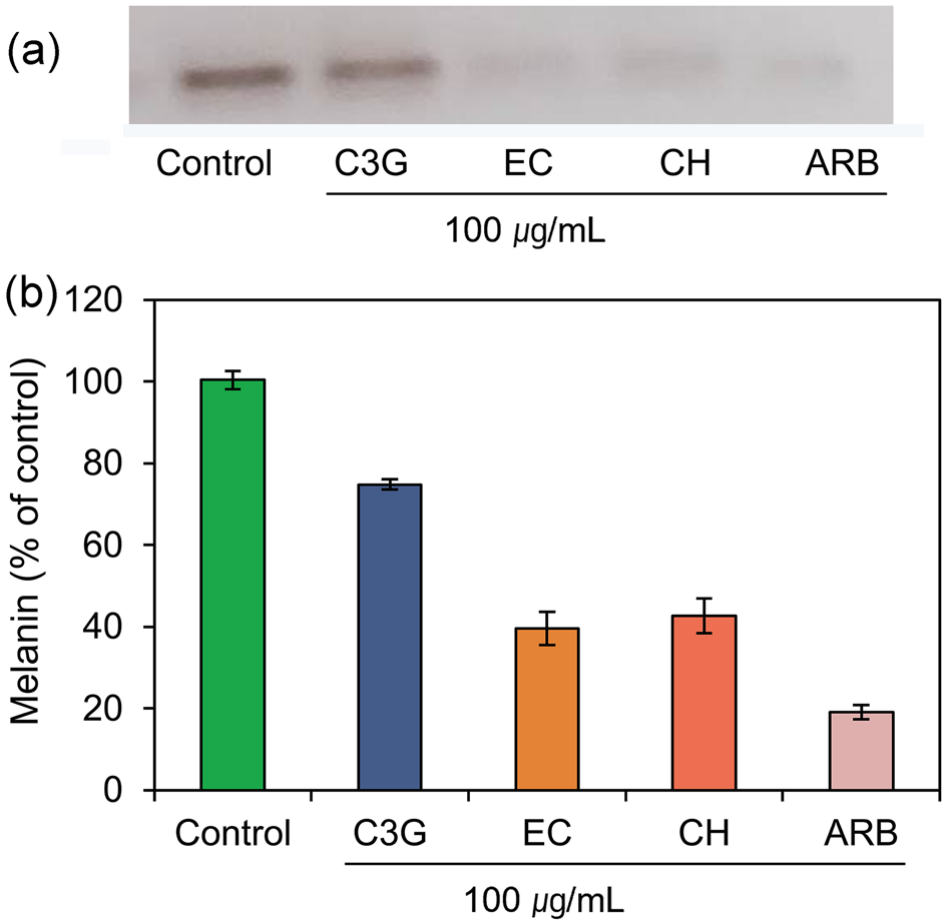
Zymogram analysis for the inhibition of the murine tyrosinase incubated with selected bioactive compounds, i.e., C3G, EC, and CH, and positive control compound, viz. ARB inhibitor at 100 µg/mL concentration. Here, (**a**) zymograms show the reduction in dark black color corresponds to the *o*-quinone production by the activity of murine tyrosinase and (**b**) measured color intensity of the produced bands with standard deviations from the triplicate experimental data.

Following, 100 μg/mL of each compound was selected to monitor the murine tyrosinase inhibition in cell-free zymography (Table S7, Figs. S18, 12). Herein, the equal number of cells were incubated with 100 μg/mL of selected flavonoids against positive control, lysed, and examined on the zymogram. Figure 12 shows no substantial reduction in the activity of the murine tyrosinase by C3G while higher inhibition for the murine tyrosinase enzyme was noted for EC and CH against ARB inhibitor and control (no treatment). These observations were in accordance with the mh-Tyr zymography where a significant reduction in enzyme activity was noted for the EC and CH (Fig. 10). Therefore, EC and CH were marked as potential inhibitors of the murine tyrosinase enzyme by comparison to C3G.

### Melanin content analysis

**Figure 13:**
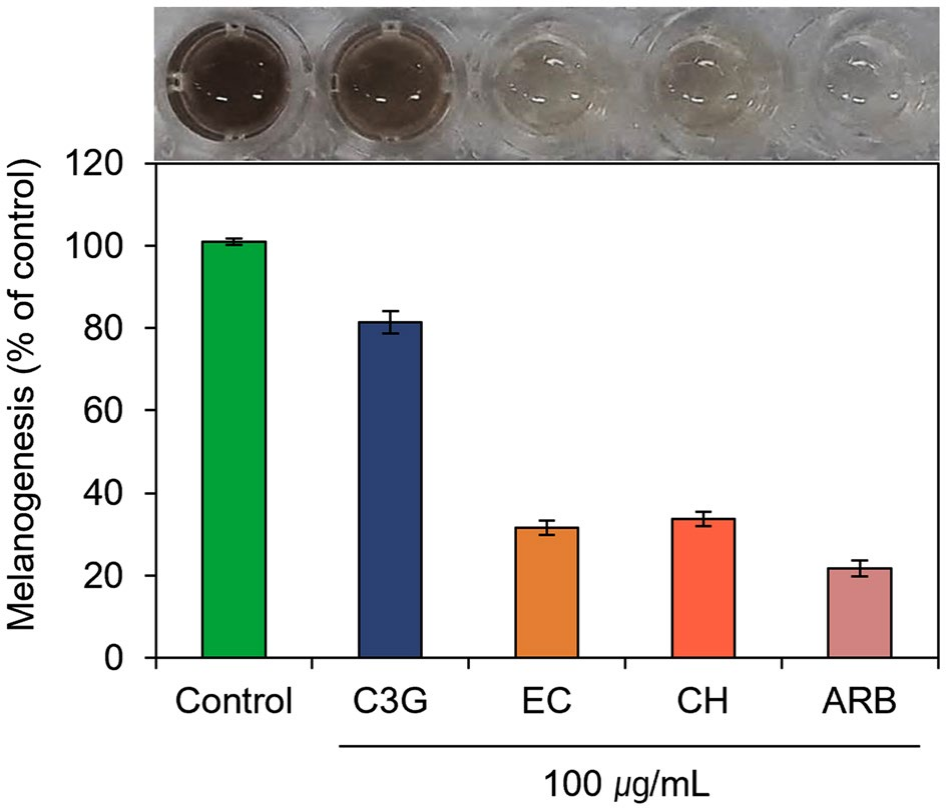
Melanin content measurement from the treated murine melanoma cells with selected bioactive compounds, i.e., C3G, EC, and CH, and positive control compound, viz. ARB inhibitor.

The reduction in melanin production by the treatment of selected chemicals was also monitored in the murine melanoma cell line B16F10 (Table S7). Figure 13 exhibits a substantial reduction in the melanin synthesis in the murine melanoma cells by the treatment of EC and CH against ARB inhibitor and control (no treatment). These results were also relatively proportional to the inhibition of murine tyrosinase enzyme activity as predicted from the cell-free tyrosinase inhibition assay (Fig. 12). Under these observations, the EC and CH were sorted as potential inhibitors for the melanin production in the murine melanoma cells, and hence, can be considerded for further hyperpigmentation treatment.

## Discussion

Among several factors responsible for human skin color, melanogenesis is a well-established pathway for melanin biosynthesis. Although ideal production of melanin is associated with an effective defense against UV radiations, abnormal melanin biosynthesis and accumulation have been linked to several dermatological disorders in humans, such as hyperpigmentation and skin cancer. In this process, tyrosinase has been determined to contribute essential function in the melanin biosynthesis via oxidation of l-tyrosine; and hence, tyrosinase is an important target for the treatment of pigmentation to develop cosmetically skin-whitening agents and therapeutics against tyrosinase linked diseases^11,23,25,26,84–86^. Mechanistically, the phenyl ring in the tyrosinase substrate was elucidated to react with copper ion (CuA) to initiate the electrophilic monooxygenation reaction on the phenol group; this is followed by an intermediate complex formation for the substrate attachment to both the copper ions (CuA and CuB) in the catalytic pocket. In the next step, such complex endures homolytic dissociation to produce the (*o*)ortho-quinone and deoxy-tyrosinase. Later, the deoxy-tyrosinase form of the enzyme unites with the oxygen to revitalize the oxy-tyrosinase form; and thus, the phenol-oxidation cycle remains until the phenol and/or oxygen are depleted in the substrate-enzyme reaction^9^. In this context, several natural products, flavonoids have been identified as tyrosinase inhibitors^25^. Of note, many of the flavonoids contain a catechol group and can be easily oxidized by tyrosinase, as revealed earlier for quercetin which functioned as a tyrosinase substrate^87,88^. Therefore, this study was designed and conducted to assess the inhibition of tyrosinase by the abundant and popular flavonoids, viz. C3G, EC, and CH, by comparison to ARB inhibitor as a positive control using computational modeling and in vitro techniques.

As mushroom tyrosinase (mh-Tyr) is commonly employed as a target enzyme to screen the potential inhibitors of melanogenesis^89^; hence, the crystal structure of mh-Tyr was considered for computational analysis with selected flavonoids in the absence of crystal structure for mammalian tyrosinase enzyme. Generally, tyrosinases exit in the form of tetramers as two sets of identical subunits (H and L)^90^, where catalytic subunit (H) comprises a binuclear copper-binding region at the core of four α-helices structures. These binuclear copper ions are connected to six histidine residues (His^61^, His^85^, His^94^, His^259^, His^263^, and His^296^ residues), which further interact with the adjacent residues, viz. Phe^90^ and Phe^292^, to acquire restricted flexibility in the side chains for the stability of the copper-binding site^37,91^. Hence, an effective and secure attachment of a ligand or inhibitor into the tyrosinase catalytic pocket involves interactions with the binuclear copper ions as well as respective coordinated histidine residues and other adjoining residues^92^.

In this study, the stringent XP docking method was used to produce the ideal docked conformations of selected compounds with mh-Tyr, which revealed highest negative docking scores (− 9.346 to − 5.795 kcal/mol) for the selected compounds. Notably, all the docked poses demonstrated substantial intermolecular contacts formation with essential residues (His^61^, His^85^, His^94^, His^259^, and His^263^) and binuclear copper active site in the mh-Tyr enzyme (Table S1, Fig. 2). Importantly, C3G exhibited metal-coordination bonds with the binuclear copper active site via oxygen atoms of the (*m*)meta-diphenols (A-ring) while EC and CH exhibited similar interactions with the mh-Tyr via oxygen atom on the (*o*)ortho-diphenols or catechol group (B-ring) (Table S1, Fig. 2). However, no such interaction was observed for the ARB inhibitor with the mh-Tyr enzyme (Fig. 2). Interestingly, the interacting residues with the selected flavonoids were known as active residues in tyrosinase^37^ and have been cited for interactions with potent tyrosinase inhibitors^92–96^. Moreover, recent studies also established that among the various types of compounds able to block melanogenesis, only specific inactivators and irreversible inhibitors of tyrosinase interacted and inhibited the tyrosinase activity^66,97^. Therefore, for true tyrosinase inhibitors, four types of the mechanism were postulated and demonstrated, such as non-competitive, competitive, uncompetitive, and mixed type (competitive/uncompetitive) inihibtion^17,28,35^. Particularly, compounds structurally mimicking the substrate of tyrosinase, such as compounds with phenolic substructures, were advocated to function as copper chelators. Importantly, the location and number of hydroxyl groups on the phenyl ring were discovered to significantly affect the tyrosinase inhibitory activity in the case of bioactive flavonoids^98^. In this context, various flavones and flavonols containing a catechol moiety in their B-ring with *o*-diphenols have been reported as strong competitive inhibitors of tyrosinase^94,99–102^, which is associated with tyrosinase inhibition^99^. Additionally, the *o*-diphenols in the B-ring of flavonoids experience slow oxidation by comparison to *m*-diphenols, i.e. A-ring^103^. This is because flavonoids with catechol groups, such as EC and CH, lacks conjugation to the 3-OH group in C-ring which shield such molecules to form (*p*)para-quinone methides, and thus, flavonoids with these structural properties restrict their oxidation at the B-ring by the tyrosinase enzyme^104^. Typically, flavonoids with catechol group in the B-ring acted as an *o*-diphenolic substrate for the oxidation by both the *oxy*-and *met*-forms tyrosinase enzyme^104^ and predicted with optimal orientation for Quintox mechanism^105^, a geometry required for inactivation of tyrosinase, as reported earlier for green tea catechins^66^. Altogether, C3G was predicted as mh-Tyr alternative substrates which exhibit rapid oxidation, and hence, served as a weak competitive inhibitor by comparison to EC and CH compounds.

Generally, protein or protein docked complexes may hold a rugged energy landscape with many accessible local minima which arises perplexity for short MD simulation to characterize the global minima^71^. Thus, as advocated by the D E Shaw group that longer simulation offers improved results to identify the global minima^75^, the best optimal binding conformation of mh-Tyr with selected flavonoids (C3G, EC, and CH) and positive control (ARB inhibitor) was studied for complex stability and molecular contact profiling as a function of 100 ns MD simulation under explicit solvent using Desmond v5.6^49^ modules of Schrödinger suite 2018-4^50^. It is important to mention that MD simulation under implicit solvent model has been marked as less reliable and detected with dissociation of ligand from the binding site in the receptor^106^. Moreover, the force field plays a critical function in MD simulation as it regulates all the intermolecular interactions in a given system^107^. Hence, each docked complex, i.e., mh-Tyr-flavonoids and mh-Tyr-ABR inhibitor, were simulated under OPLS-2005 force field with explicit (TIP4P) water solvent for 100 ns interval. Among the generated MD trajectories, significant stability or global minima and interactions were observed for the docked C3G in the active pocket of the mh-Tyr against EC, CH, and ARB inhibitor (Figs. 5, 6); these results emphasize that C3G have substantial interactions with the catalytic core of the mh-Tyr enzyme through A-ring and should rapidly be oxidized by the mh-Tyr against other selected flavonoids, i.e., EC and CH, as predicted from docked poses conformation analysis (Fig. 2).

Moreover, essential dynamics assessment, commonly applied to collect and understand the functional movements in the structure of protein via collecting PCs^62^, on the respective MD trajectories revealed substantial compact residual fluctuation in docked mh-Tyr with flavonoids or ARB inhibitor against apo-mh-Tyr structure (Fig. 7). These observations correspond to the oxidation of docked flavonoids by the mh-Tyr as predicted earlier from the analysis of intermolecular interactions in docked poses and the MD simulation trajectories (Figs. 2, 5, 6). Moreover, to completely abrogate the inaccuracy and inefficiency of the screened inhibitors, end-point free energy calculations are usually computed on MD trajectory in structure-based drug design^74^. Among the different available methods, MM/GBSA method linked with MD simulations provides a good balance between computational efficiency and accuracy to compute the binding free energy^74^. Herein, mh-Tyr-C3G complex was recognized with the most considerable free binding energy before (− 34.72 kcal/mol) and after (− 74.51 ± 20.49 kcal/mol) against other bioactive compounds and positive inhibitors docked with mh-Tyr (Fig. 8). As C3G exhibited strong interaction by A-ring against other bioactive compounds, B-ring (Figs. 2, 5, 6), the calculated binding free energy again indicates the rapid oxidation of C3G against EC and CH compounds.

Furthermore, inhibition activity of the selected compounds, i.e., C3G, EC, CH, and ARB inhibitor, against mh-Tyr was also assessed using both spectrophotometric and zymography methods. Intriguingly, both the experimental observations showed contradicting results where C3G was noted for maximum mh-Tyr inhibition using spectrophotometer method while EC and CH exhibit superior results for mh-Tyr inhibition activity in zymograms (Figs. 9, 10). Notably, flavonoids are reported for chelation with copper ions in the enzyme and then irreversibly inactivate the tyrosinase enzyme^108^. Furthermore, the oxidation of flavonoids was also studied to produce byproducts, like intermediate adducts and polymers, with a large absorption spectrum in the range of 300–600 nm^109,110^. For instance, catechins hold either a catechol ring or conjugated phenol group in the B and C-rings, which can react with *o*-quinones (e.g., dopaquinone) generated by tyrosinase enzyme via two-electron redox reaction^104^. Besides, phenol groups in flavonoids were also predicted to form conjugates with *o*-quinones via a nucleophilic addition reaction, such as in quercetin^111^. Therefore, the substantial differences between the spectrophotometric and zymography calculations obtained in this study can be justified on the basis that the absorption spectrum of the byproducts generated from the oxidation of flavonoids intersects with the absorption spectra of dopachrome produced by tyrosinase; and hence, interfered with the enzyme inhibition assessment monitor via tyrosinase activity using the spectrophotometric method^104^. Moreover, in addition to direct enzyme oxidation reaction, pseudo results in absorbance may be caused by supplementary reactions taking place in the reaction mixture^104^. For instance, under l-DOPA as substrate in the reaction mixture, flavonoids with a catechol or conjugated phenol groups in B and C-ring can be oxidized by dopaquinone, where l-DOPA served as a redox shuttle between the flavonoids and the tyrosinase enzyme^104^. Thus, the spectrophotometer method to determine the functional activity of mh-Tyr treated with flavonoids and other compounds holding strong reducing or nucleophilic groups was also discussed as an inappropriate approach^104^. However, zymography overruled interferences observed in the spectrophotometric method where inhibition of the enzyme can be classified based on color band formation corresponding to the activity of an enzyme. Presumably, tyrosinase inhibition by flavonoids is described based on their capability to chelate with binuclear copper ions in the active center of the enzyme via catechol group (B-ring). In this study, the computational analysis revealed that only EC and CH were noted for such interactions while C3G established the chelation via A-ring. Moreover, protection of unconjugated 3-OH group in the C-ring with catechol group by a large group (e.g., by glycosylation or alkylation) has been linked with no enzyme inhibition, as it has been noted for quercetin 3-*O*-glucoside^112^. Similar findings were also calculated for the flavonoids secluded from the persimmon *Diospyros kaki*, where only kaempferol and quercetin exhibited strong tyrosinase inhibition while hyperoside and quercetin 3-glycosides isoquercitrin were noted for no inhibition of the tyrosinase enzyme^113^. Thus, zymogram analysis of treated mh-Tyr enzyme with selected flavonoids suggested the EC and CH are potent alternative substrates and may act as competitive inhibitors of Tyrosinase by comparison to C3G and ARB inhibitor, and similar predictions were predicted from the computational analysis.

Of note, tyrosinase inhibitors identified using mh-Tyr inhibition were noted with least or no mammalian tyrosinase inhibition, because of significant difference in the catalytic activities and substrate specificities of mh-Tyr against mammalian Tyrosinase enzyme^63,114^. Thus, selected flavonoids, i.e., C3G, EC, and CH, are of great candidature for appraising their cell viability and melanogenesis inhibition in mammalian cell system. Therefore, in vitro assessment of the selected flavonoids, viz. C3G, EC, and CH, exhibit no substantial cytotoxicity (100 μg/mL) on murine melanoma cells marking them as ideal candidates for the assessment of mammalian tyrosinase inhibition by comparison to ARB inhibitor (Fig. 11). Following, murine tyrosinase zymography for each selected compound (100 μg/mL) revealed EC and CH as potent tyrosinase inhibitors by comparison to C3G and ARB inhibitor (Fig. 12). Furthermore, the treated murine melanocytes were also noticed with a substantial reduction in melanin production levels when treated with EC and CH; yet a decrease in melanin content was also observed in the treatment with C3G at the same concentration (100 μg/mL) (Fig. 13). A recent study using murine melanoma cell culture also exhibited reduced melanogenesis by the treatment of polyhydroxy flavones^115^. Moreover, a similar experiment with murine melanoma cells also marked the polymethoxy flavones to inhibit the melanogenesis process^116^. These observations suggested that screened compounds, EC and CH, have structural resemblance with that of tyrosinase substrates, such as l-DOPA or l-tyrosinase; and hence, can displace them with their catechol group, which then attaches with the binuclear copper ions in the catalytic site of tyrosinase^88^. Collectively, these results indicate that both EC and CH could specifically inhibit both the mh-Tyr and murine tyrosinase enzyme; and hence, are highly efficient flavonoids for the tyrosinase inhibition.

## Conclusion

In this study, we have predicted the binding interaction and inhibitory activity for C3G, EC, and CH with mh-Tyr using computational and in vitro methods. Analysis of molecular docked poses revealed the substantial role of chelation via A-ring and blockage of 3-OH by glycosylation on unconjugated C-ring with catechol group in C3G compound accounts for low tyrosinase inhibition. Likewise, substantial interactions with the copper ions by catechol group (B-ring) and free 3-OH (hydroxyl) group on the unconjugated C-ring plays a key role in the strong tyrosinase inhibition. The docked flavonoids were also noted for additional substantial intermolecular interactions with the hydrophobic and aromatic residues around the catalytic site of mh-Tyr structure. Furthermore, explicit solvent model and binding free energy calculations suggested the stability and dynamic behaviors of respective docked complexes. Interestingly, the observations extracted from computational analysis were in accordance with the calculated in vitro enzyme inhibition activities, i.e., stronger interaction of C3G (glycosylation of 3-OH group) via A-ring with binuclear copper ion results in its complete oxidation by the enzyme against EC and CH compounds. Hence, this study advocates that computational analysis can be used in the first instance when assessing the ability of a compound as a tyrosinase inhibitor to save resources and time; this should be followed by validation using zymography to avoid the pseudo positive hits. Moreover, a detail mechanism can be performed using QM/MM simulation to understand the proton transfer during oxidation of selected flavonoids when interacting through A-ring and B-ring in the presence of blocked or free 3-OH group on the unconjugated C-ring with catechol group. Importantly, as results collected from spectrophotometer method were established to be not true for the selected flavonoids, it also demands improved and advanced rapid biophysical techniques to perform the enzyme kinetics for tyrosinase and similar enzymes with flavonoids. Our study provides a base for the screening and validation of true tyrosinase inhibitors for treating tyrosinase linked disorders and diseases.

## Supplementary Information

Supplementary Information 1.

Supplementary Movie 1.

Supplementary Movie 2.

Supplementary Movie 3.

Supplementary Movie 4.
