# Supplementary material for: Determination of tyrosinase-cyanidin-3-O-glucoside and (−/+)-catechin binding modes reveal mechanistic differences in tyrosinase inhibition
Source: Sci Rep. 2021 Dec 30;11:24494. doi: 10.1038/s41598-021-03569-1 (PMC8718538; doi:10.1038/s41598-021-03569-1)
Supplement: Supplementary file 1 — Supplementary Information 1. [file 41598_2021_3569_MOESM1_ESM.docx]

**Supplementary Information**

**Determination of tyrosinase-cyanidin-3-*O*-glucoside and (-/+)-catechin binding modes reveal mechanistic differences in tyrosinase inhibition**

^1,4^ Kyung Eun Lee**^†*^**, ^1^Shiv Bharadwaj**^†*^**, ^2^Amaresh Kumar Sahoo**^*^**, ^3^Umesh Yadava**^*^**, and ^1^Sang Gu Kang*****

^1^Department of Biotechnology, Institute of Biotechnology, College of Life and Applied Sciences, Yeungnam University, 280 Daehak-Ro, Gyeongsan, Gyeongbuk 38541, Korea

^2^Department of Applied Sciences, Indian Institute of Information Technology Allahabad, Allahabad 211015, Uttar Pradesh, India

^3^Department of Physics, Deen Dayal Upadhyay Gorakhpur University, Gorakhpur, India.

^4^Stemforce, 313 Institute of Industrial Technology, Yeungnam University, 280 Daehak-Ro, Gyeongsan, Gyeongbuk 38541, Korea

**^†^**These authors contributed equally to this work.

***Corresponding authors**

K.E.L: keun126@ynu.ac.kr; S.B: shiv@ynu.ac.kr; A.K.S: [asahoo@iiita.ac.in](mailto:asahoo@iiita.ac.in); U.Y: u_yadava@yahoo.com; S.G.K: kangsg@yu.ac.kr

**S1. Results**

**S1.1. Ligands and receptor crystal structure collection**

**
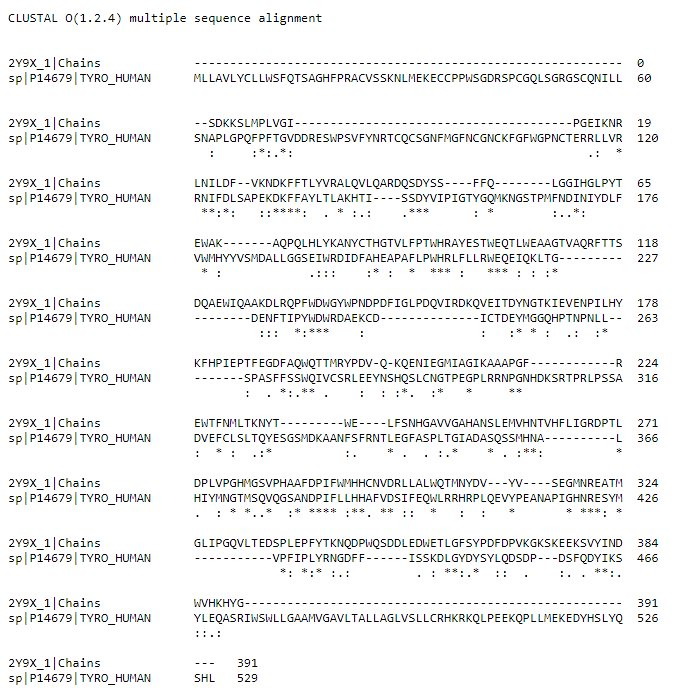
**

**Figure S1.** Sequence alignment of human tyrosinase (TYRO_HUMAN) with the mushroom tyrosinase (2Y9X_1) using Clustral W multiple sequence alignment programme.

**
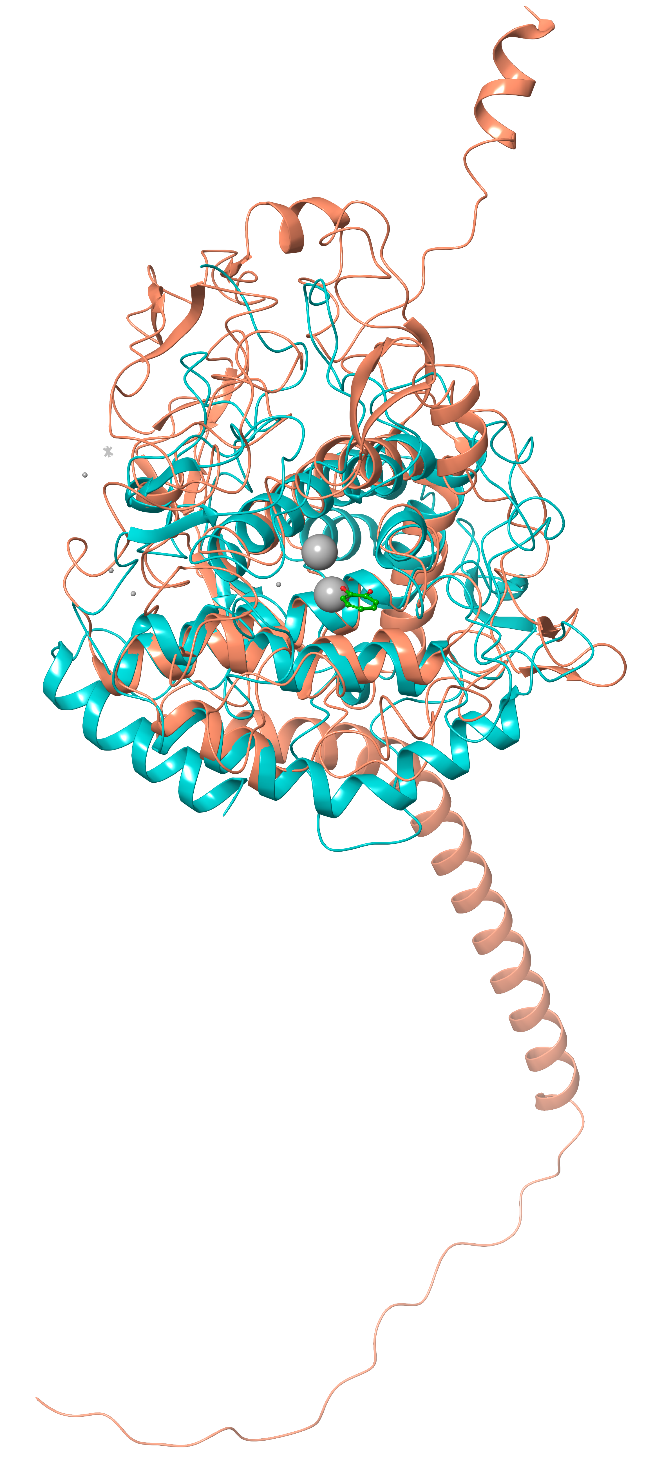
**

**Figure S2.** 3D structure alignment of human tyrosinase protein (Orange color predicted by AlphaFold programme) with the crystal structure of mushroom tyrosinase (cyan color) generated using free academic Schrödinger-Maestro v12.6 suite ^1^; URL: <https://www.schrodinger.com/freemaestro>. Herein, grey color sphere represents the copper ions while co-crystallized tropolone inhibitor is presented with green color in the 3D crystal structure of tyrosinase from *Agaricus bisporus* mushroom (PDB ID: 2Y9X) ^2^.

**
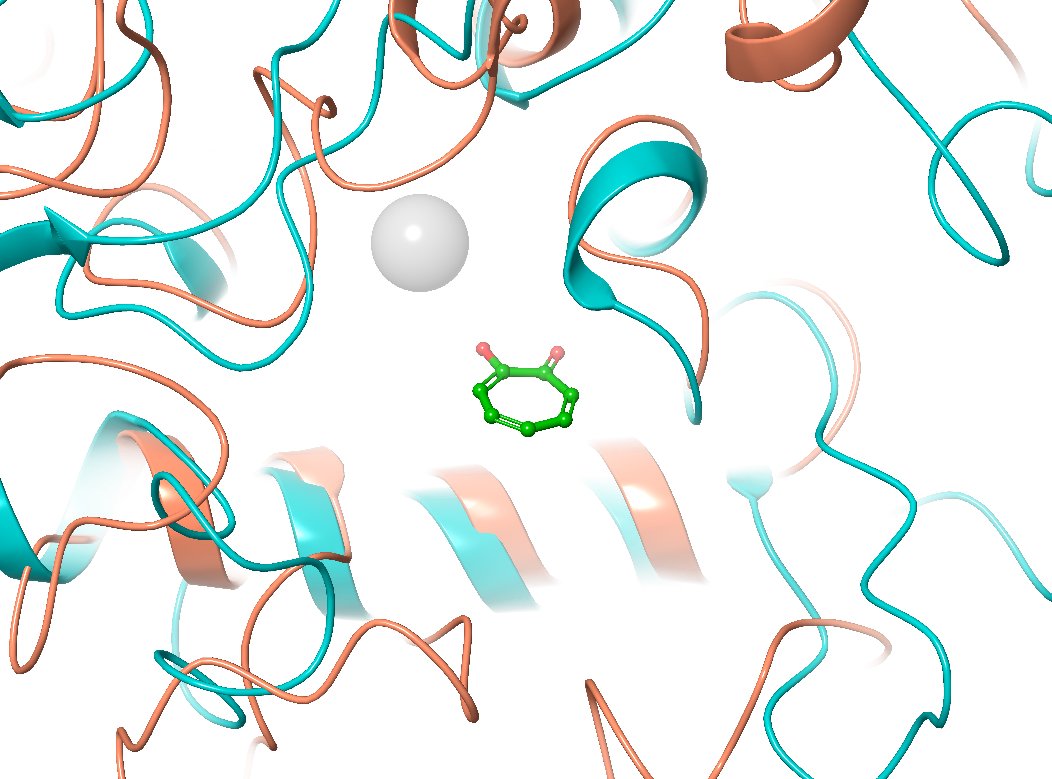
**

**Figure S3:** 3D structure alignment of catalytic pocket of human tyrosinase protein (Orange color predicted by AlphaFold programme) with the crystal structure of mushroom tyrosinase (cyan color) generated using free academic Schrödinger-Maestro v12.6 suite ^1^; URL: <https://www.schrodinger.com/freemaestro>. Herein, grey color sphere represents the copper ions while co-crystallized tropolone inhibitor is presented with green color in the 3D crystal structure of tyrosinase from *Agaricus bisporus* mushroom (PDB ID: 2Y9X) ^2^

**S1.2. Molecular docking and intermolecular interaction analysis**

**
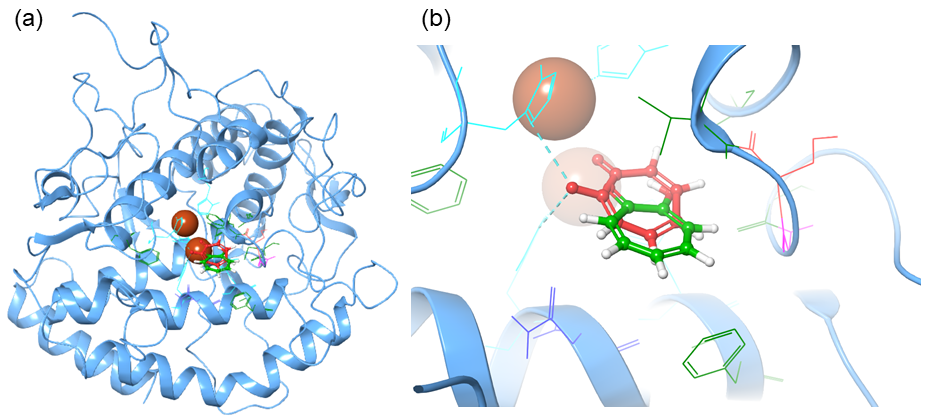
**

**Figure S4:** Alignemnet of the mh-Tyr 3D crystal structure with the respective re-docked complex, where green color ligand represents the co-crystallized tropolone inhibitor conformation and red color ligand shows the re-docked conformation of tropolone inhibitor in the active pocket of mh-Tyr structure; (a) depicts the complete aligned 3D structures while (b) shows the zoomed active pocket containing the ligands with intermolecule interactions. All the images were generated using free academic Schrödinger-Maestro v12.6 suite ^1^; URL: <https://www.schrodinger.com/freemaestro>.


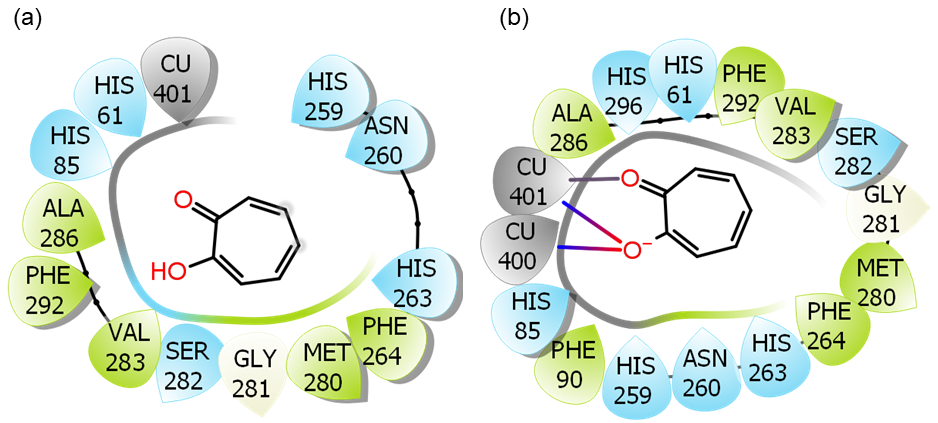


**Figure S5**. 2D interaction poses for the mh-Tyr protein with tropolone inhibitor in (a) co-cyrstalised conformation and (b) re-docked conformation using XP docking protocol. Herein, hydrophobic (green), polar (blue), glycine (grey), metal coordination bond (black line), and salt bridge (red-violet line) interactions are depicted in the respective complexes. All the images were generated using free academic Schrödinger-Maestro v12.6 suite ^1^; URL: <https://www.schrodinger.com/freemaestro>.

**Table S1:** List of intermolecular interactions noted in the docked complexes of mh-Tyr with selected bioactive compounds using extra precision molecular docking protocol.

| S.no. | Compounds | Docking score  (kcal/mol) |  | Intermolecular interactions | | | | | | | |
| --- | --- | --- | --- | --- | --- | --- | --- | --- | --- | --- | --- |
|  |  |  |  | H-bond | Salt bridge/  *Metal coordination | Hydrophobic | π- π /  *π-cation | Polar | Positive | Negative | Glycine |
| 1 | C3G | -9.346 |  | Gly^281^, Arg^268^,  Glu^322^(2) | Cu^400^, Cu^401^ | Val^248^, Phe^264^, Val^283^ | His^85^, His^263^ | His^61^, His^85^, Hie^244^, His^259^, Asn^260^, His^263^, Ser^282^ | Arg^268^ | Glu^322^ | Gly^281^ |
| 2 | EC | -6.595 |  | -- | *Cu^400^ | Phe^90^, Cys^83^,Val^248^, Phe^264^, Met^280^ , Val^283^, Ala^286^, Phe^292^ | His^85^, His^259^, His^263^ | His^61^, His^85^, His^244^, His^259^, Asn^260^, His^263^, Ser^282^ | -- | -- | Gly^281^ |
| 3 | CH | -5.301 |  | Asn^260^, Gly^281^ | Cu^400^, Cu^401^,  *Cu^400^, *Cu^401^ | Phe^90^,Val^248^, Phe^264^, Pro^277^, Met^280^, Val^283^, Ala^286^, Phe^292^ | His^259^, His^263^, Phe^264^, *Arg^268^ | His^61^, His^85^, His^94^, His^244^, His^259^, Asn^260^, His^263^, Ser^282^, His^296^ | Arg^268^ | Glu^256^ | Gly^281^ |
| 4 | ARB | -5.795 |  | Asn^260^ | -- | Phe^90^, Val^248^, Met^257^, Phe^264^, Met^280^,Val^283^, Ala^286^, Phe^292^ | Phe^264^ | His^61^, His^85^, Hie^244^, His^259^, Asn^260^, His^263^, Ser^282^ | -- | Glu^256^ | Gly^281^ |

**S1.2. Molecular dynamics simulation analysis**


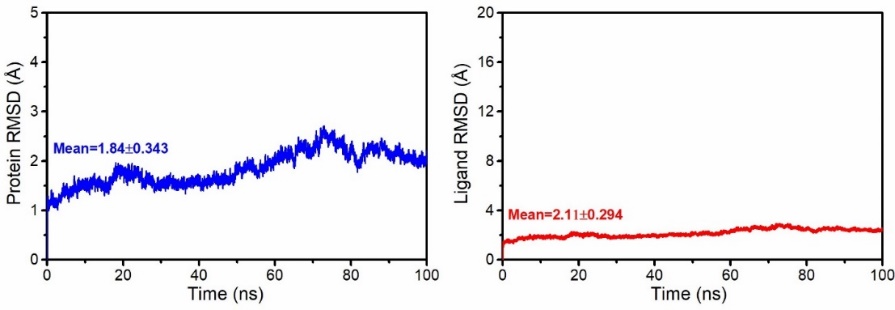


**Figure S6**: RMSD values plotted for the docked mh-Tyr protein and mh-Tyr fit reference ligands, i.e. tropolone inhibitor, extracted from the respective simulated complex with respect to 100 ns MD simulation.

**Figure S7:** RMSF analysis for the docked mh-Tyr protein with reference ligands, i.e. tropolone inhibitor, extracted from the 100 ns MD simulation trajectory.


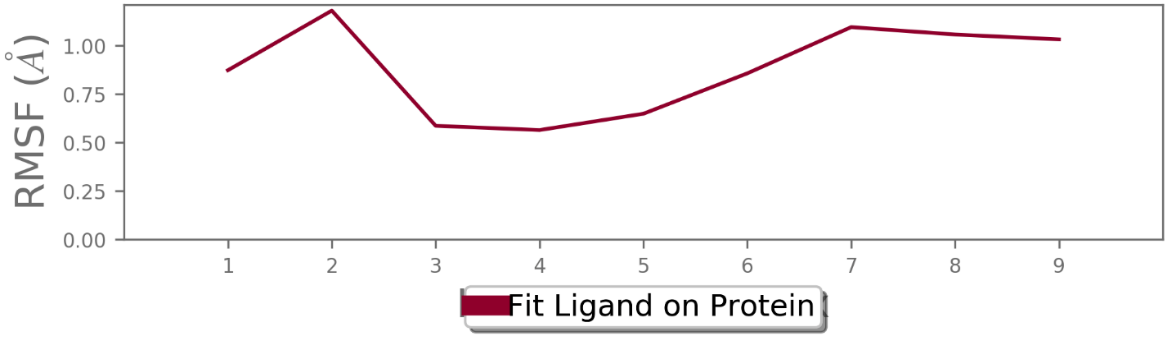


**Figure S8:** RMSF analysis for the protein fit ligands, i.e. tropolone inhibitor, as reference inhibitor, extracted from the respective simulated complexes as function of 100 ns simulation interval.

**S1.2.1. Last pose molecular contact profiling**

**Table S2**: List of molecular contact formation between the docked bioactive compounds and residue in the active pocket of mh-Tyr logged from the extracted last poses after 100 ns molecular dynamics simulation.

| S.no. | Compounds |  | Intermolecular interactions | | | | | | | |
| --- | --- | --- | --- | --- | --- | --- | --- | --- | --- | --- |
|  |  |  | H-bond | Hydrophobic | π-π/*π-cation | Salt bridge/  *Metal coordination | Polar | Positive | Negative | Glycine |
| 1 | C3G |  | Asn^260^, His^263^ | Met^257^, Phe^264^, Met^280^, Val^283^, Phe^292^ | His^85^, *His^85^,  His^263^ | *Cu^400^, *Cu^401^ | His^61^, His^85^, His^94^, Asn^243^, Hie^244^, Asn^260^, His^263^, His^296^ | -- | Glu^322^, Glu^256^ | Gly^86^, |
| 2 | EC |  | Gly^281^ | Phe^90^, Val^248^, Phe^264^, Val^283^, Ala^286^, Phe^292^ | -- | *Cu^400^, *Cu^401^ | His^61^, His^85^, His^244^, His^259^, His^260^, His^263^, Ser^282^, His^296^ | -- | -- | Gly^281^ |
| 3 | CH |  | Ile^266^, Gly^281^ | Leu^265^, Ile^266^, Val^283^, Ala^286^, Phe^292^ | -- | *Cu^400^(2), *Cu^401^(2) | His^61^, His^85^, His^259^, His^263^, Ser^282^, His^296^ | Arg^268^ | -- | Gly^281^, Gly^267^ |
| 4 | ARB |  | Val^283^, Ala^286^ | Tyr65, Val^283^, Pro^284^, Ala^286^, Ala^287^, Phe^292^ | -- | *Cu^401^ | His^61^, His^85^, His^263^, His^285^, His^296^ | -- | Glu^256^ | Gly^62^ |

**S1.4. Root-mean square deviation and fluctuation analysis**





**Figure S9**: RMSD analysis for the apo-protein extracted from the MD simulation trajectory as function of time for 100 ns simulation interval.

**MD movie S1**: MD simulation movie rendered for the mh-Tyr-C3G docked complex using 10,000 snapshots obtained during 100 ns interval.

**MD movie S2**: MD simulation movie rendered for the mh-Tyr-EC docked complex using 10,000 snapshots obtained during 100 ns interval.

**MD movie S3**: MD simulation movie rendered for the mh-Tyr-CH docked complex using 10,000 snapshots obtained during 100 ns interval.

**MD movie S4**: MD simulation movie rendered for the mh-Tyr-ARB inhibitor docked complex using 10,000 snapshots obtained during 100 ns interval.


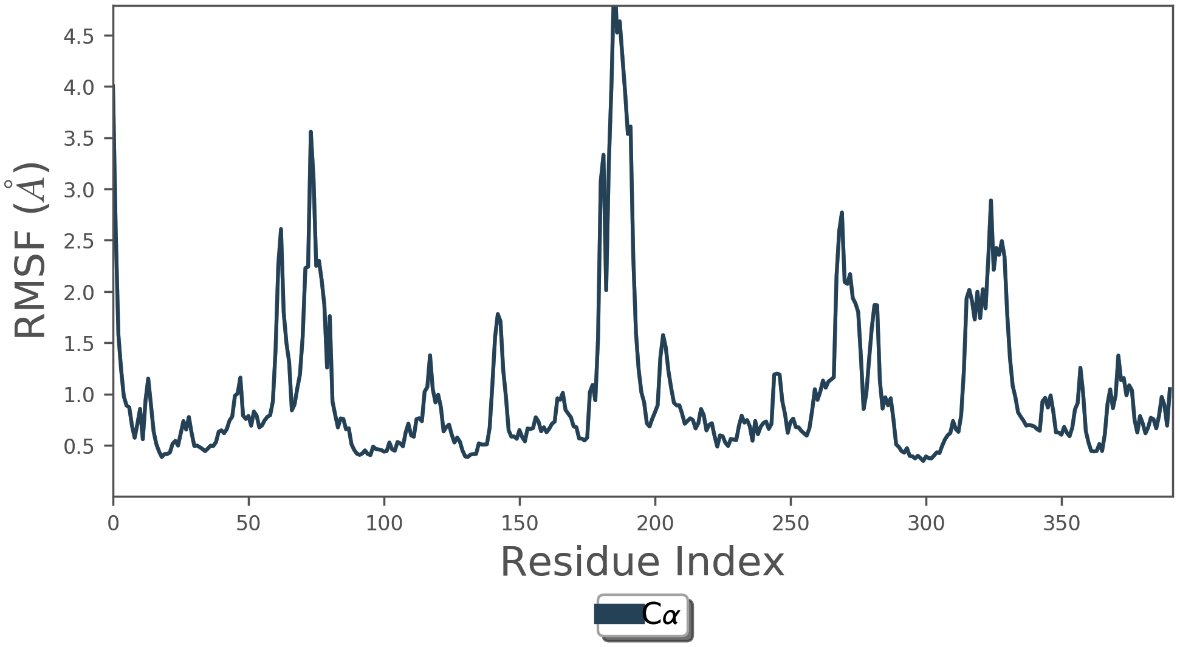


**Figure S10:** RMSF analysis for the apo-protein structure of mh-Tyr extracted from the 100 ns MD simulation trajectory.


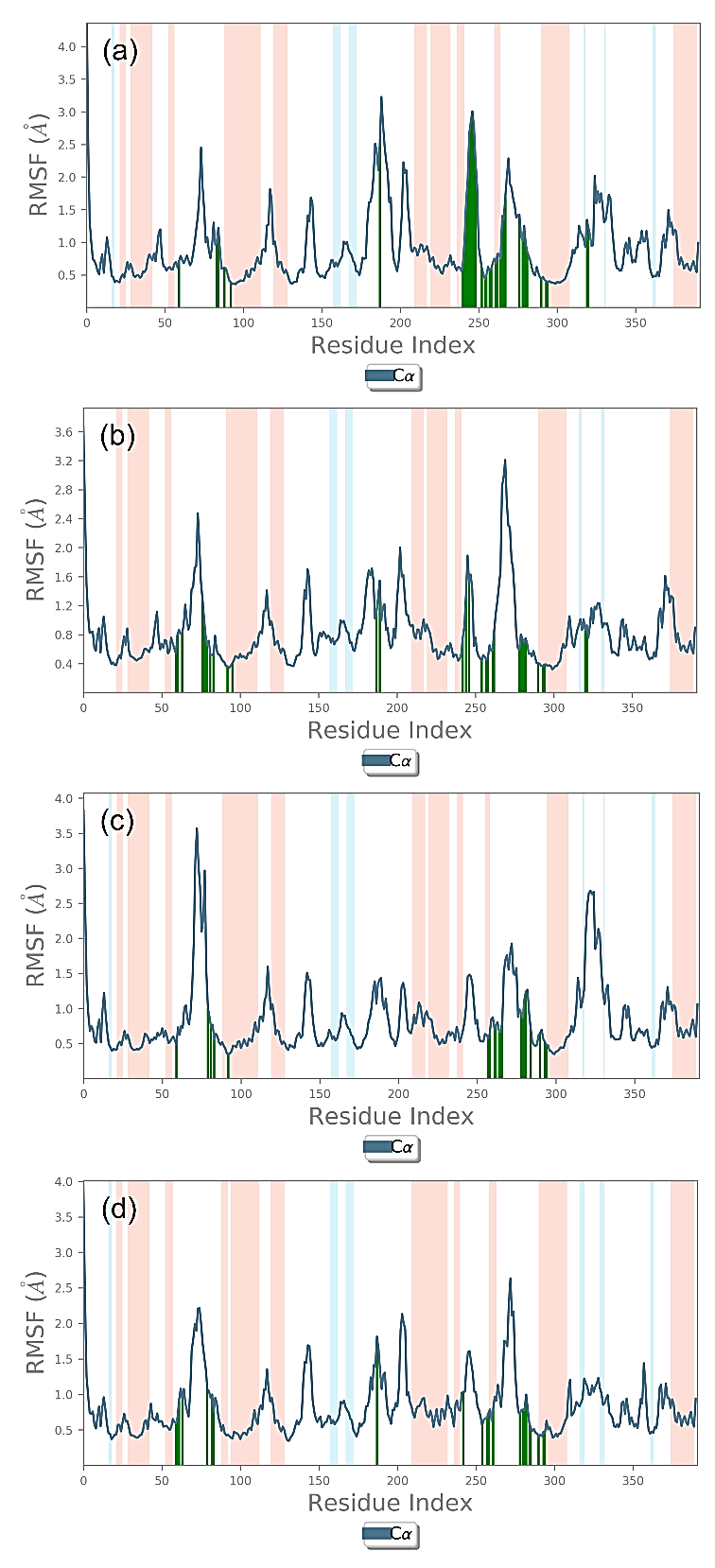


**Figure S11:** RMSF for the protein structure extracted from the simulation trajectories of docked complexes, i.e., (a) Cyanidine-3-O-gucoside, (b) Epicatechin, (c) Catechin, and (d) Arbutin as positive inhibitor, as a function of 100 ns simulation interval.


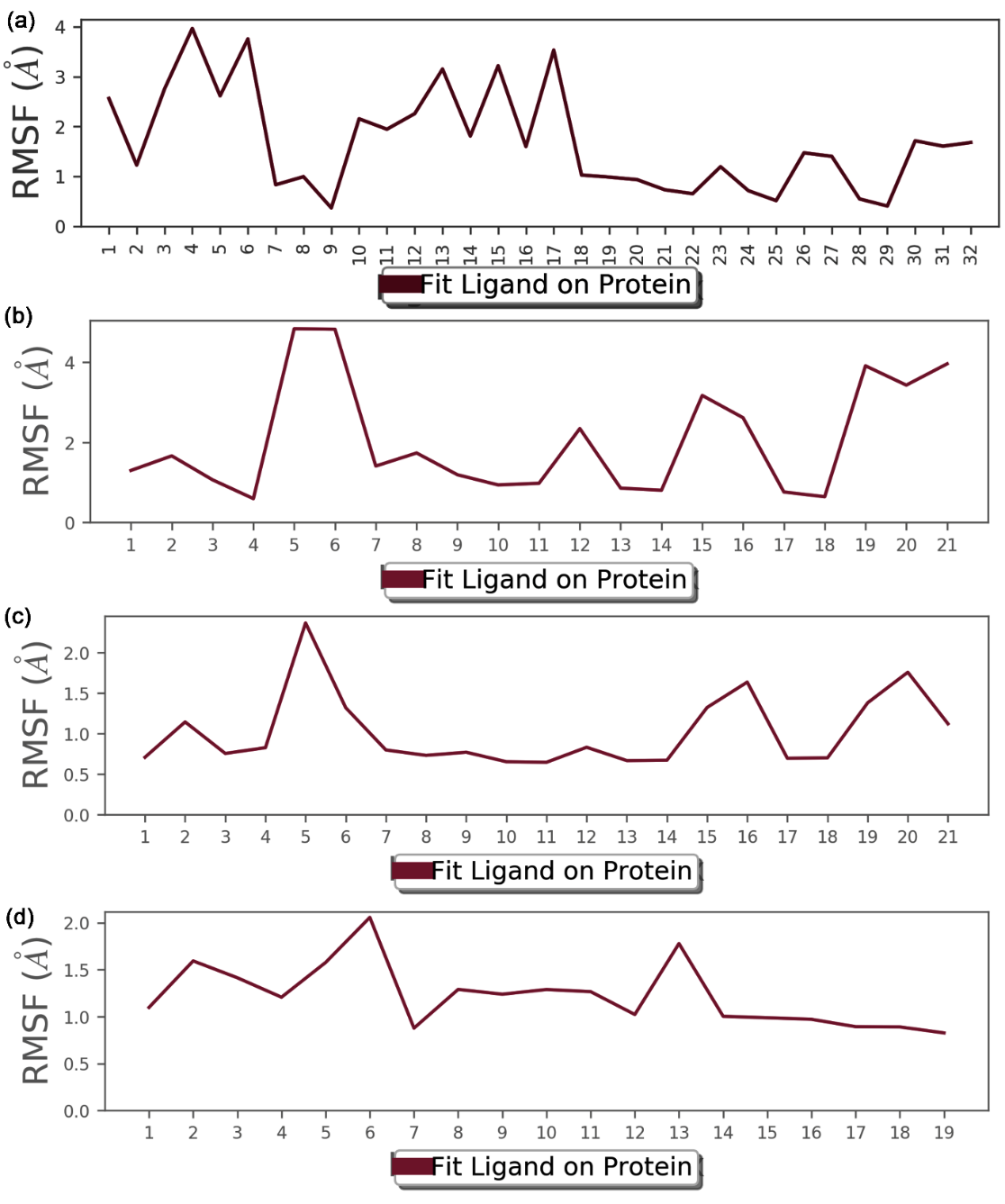


**Figure S12:** RMSF analysis for the protein fit ligands, i.e., (a) Cyanidine-3-O-gucoside, (b) Epicatechin, (c) Catechin, and (d) Arbutin as positive inhibitor, extracted from the respective simulated complexes as function of 100 ns simulation interval.


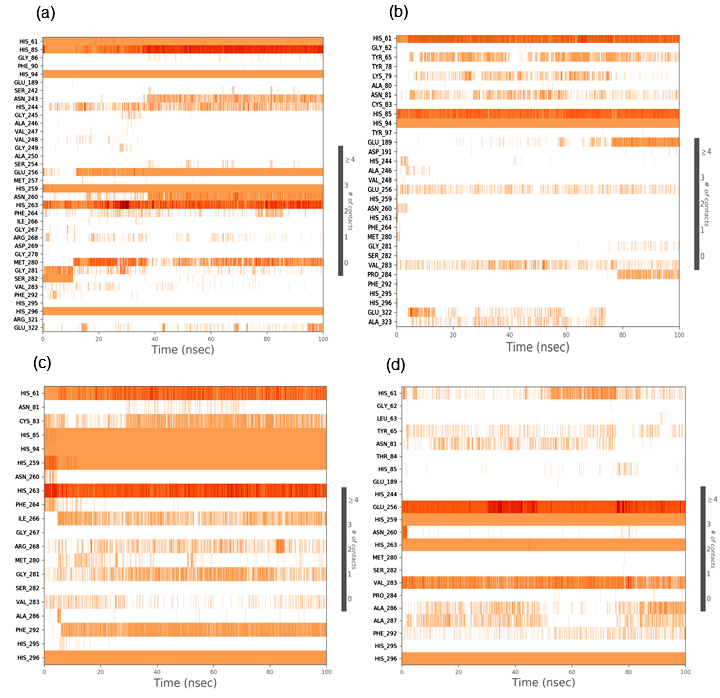


**Figure S13**: Total intermolecular interaction profiling (darker shade of orange indicates more than one contact on that frame with the residue) for the docked complexes (a) C3G, (b) EC, (c) CH, and (d) ARB with the active residues in the catalytic pocket of mh-Tyr were extracted during 100 ns MD simulation interval. These images were rendered using the SID module in the free academic Desmond v5.6 ^3^; URL: <https://www.deshawresearch.com/resources_desmond.html>.

**S1.5. Net binding free energy analysis**

**Table S3**: Comparison of MM/GBSA binding free energy and respective contributing energy components in initial docked poses against respective extracted poses from last 10 ns MD trajectories.

| MMGBSA  components | C3G | | EC | | CH | | ABR |  |
| --- | --- | --- | --- | --- | --- | --- | --- | --- |
|  | Initial Pose | MD poses | Initial Pose | MD poses | Initial Pose | MD poses | Initial Pose | MD poses |
| ΔG Bind | -34.72 | -74.51±20.49 | 12.84 | -2.67±7.03 | 3.1 | -3.68±3.47 | -7.23 | -31.09±8.76 |
| ΔG Bind Coulomb | -36.64 | -1103.01±220.53 | -18.84 | -20.49±2.51 | -41.63 | -36.43±4.72 | -27.29 | -34.33±4.4 |
| ΔG Bind Covalent | 6.47 | 5.88±1.68 | 2.95 | 4.24±1.31 | 8.98 | 2.36±0.68 | 7.94 | 6.74±1.36 |
| ΔG Bind Hbond | -2.95 | -7.78±1.73 | -1.28 | -1.46±0.29 | -1.23 | -1.05±0.19 | -1.57 | -3.22±0.56 |
| ΔG Bind Lipo | -9.59 | -8.07±0.76 | -17.06 | -8.25±1.56 | -10.59 | -7.9±0.49 | -13.49 | -11.72±1.07 |
| ΔG Bind Packing | -5.69 | -6.78±0.64 | -4.08 | -2.67±0.49 | -4.72 | -3.2±0.39 | -1.53 | -0.43±0.58 |
| ΔG Bind selfCont | 0 | 0±0 | 0 | 0±0 | 0 | 0±0 | 0 | 0±0 |
| ΔG Bind Solv GB | 50.83 | 1105.81±186.65 | 75.34 | 49.28±3.65 | 74.33 | 70.32±2.98 | 52.58 | 43.11±4.83 |
| ΔG Bind vdW | -37.15 | -60.54±17.17 | -24.19 | -23.32±3.06 | -22.04 | -27.78±1.86 | -23.86 | -31.23±2.71 |
| Lig Strain Energy | 7.79 | 47.91±17.76 | 4.33 | 4.34±1.4 | 11.08 | 2.87±1.13 | 8.77 | 6.85±1.72 |

**S1.6. Mushroom tyrosinase inhibition assay**

**Table S4**: The mh-Tyr inhibition analysis using spectrophotometer method against the different concentrations of selected bioactive compounds by comparison to positive control.

| mh-Tyr inhibition (%) | Compounds |  | Concentration (μg/mL) | | | | |
| --- | --- | --- | --- | --- | --- | --- | --- |
|  |  |  | 10 | 50 | 100 | 500 | 1000 |
|  | C3G |  | 11.9 | 39.7 | 66.9 | 75.1 | 83.2 |
| AVERAGE | EC |  | 2.1 | 2.9 | 4.5 | 10.2 | 12.1 |
|  | CH |  | 1.8 | 5.1 | 8.3 | 12.8 | 15.4 |
|  | ARB |  | 18.4 | 20.7 | 25.7 | 42.5 | 65.2 |
| STDEV | C3G |  | 3.93 | 2.69 | 0.66 | 1.56 | 3.95 |
|  | EC |  | 2.71 | 1.25 | 1.63 | 3.12 | 2.71 |
|  | CH |  | 1.63 | 3.60 | 2.17 | 2.56 | 3.53 |
|  | ARB |  | 1.13 | 0.98 | 0.76 | 1.67 | 0.76 |
| *t*-test | C3G |  | 0.033 | 0.131 | 0.301 | 0.056 | 0.096 |
|  | EC |  | 0.037 | 0.095 | 0.159 | 0.084 | 0.072 |
|  | CH |  | 0.131 | 0.076 | 0.371 | 0.069 | 0.008 |
|  | ARB |  | 0.033 | 0.131 | 0.301 | 0.056 | 0.096 |

**Table S5**: The mh-Tyr zymography analysis against the different concentrations of selected bioactive compounds by comparison to positive control.

| mh-Tyr zymogram analysis  (% of control) | Compounds |  | Concentration (μg/mL) | | | | | |
| --- | --- | --- | --- | --- | --- | --- | --- | --- |
|  |  |  | 0 | 10 | 50 | 100 | 500 | 1000 |
|  | C3G |  | 100.0 | 80.6 | 66.5 | 64.3 | 63.0 | 63.2 |
| AVERAGE | EC |  | 100.0 | 97.9 | 88.8 | 77.5 | 17.9 | 3.9 |
|  | CH |  | 100.0 | 86.8 | 60.5 | 58.2 | 27.3 | 21.5 |
|  | ARB |  | 100.0 | 75.9 | 69.6 | 65.9 | 30.4 | 28.4 |
| STDEV | C3G |  | -- | 2.37 | 1.18 | 1.26 | 4.43 | 2.47 |
|  | EC |  | -- | 3.57 | 3.73 | 4.56 | 2.00 | 2.37 |
|  | CH |  | -- | 1.65 | 0.86 | 5.40 | 1.63 | 2.42 |
|  | ARB |  | -- | 1.34 | 1.39 | 2.51 | 2.44 | 0.66 |
| *t*-test | C3G |  | -- | 0.055 | 0.016 | 0.016 | 0.054 | 0.030 |
|  | EC |  | -- | 0.120 | 0.042 | 0.049 | 0.012 | 0.015 |
|  | CH |  | -- | 0.323 | 0.035 | 0.107 | 0.009 | 0.011 |
|  | ARB |  | -- | 0.025 | 0.021 | 0.033 | 0.016 | 0.004 |


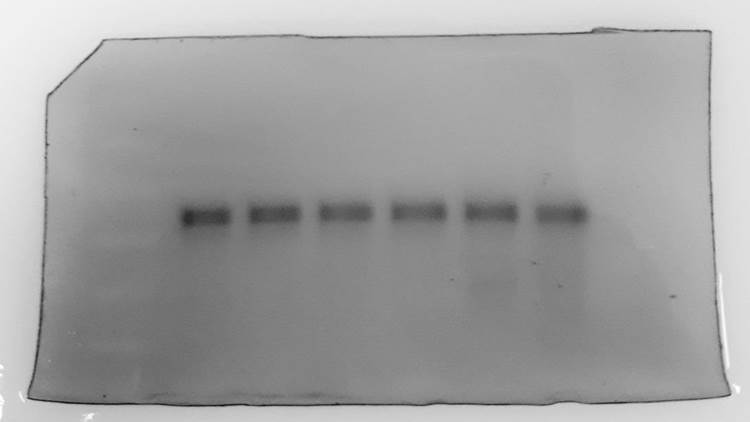


**Figure S14.** Zymograms analysis for the inhibition of the mh-Tyr enzyme incubated with different concentrations of C3G compound (10-1000 µg/ml).


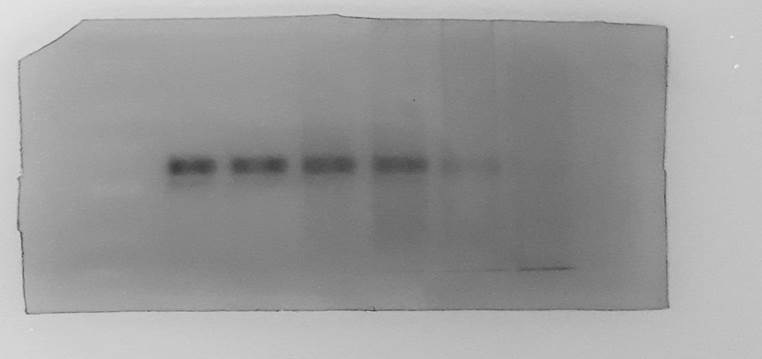


**Figure S15.** Zymograms analysis for the inhibition of the mh-Tyr enzyme incubated with different concentrations of EC compound (10-1000 µg/ml).


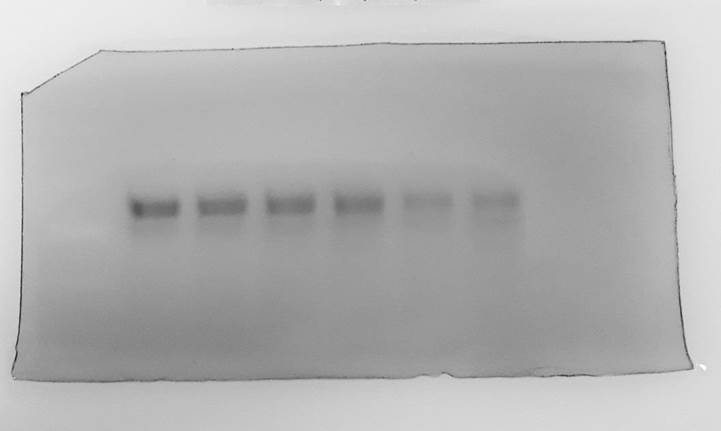


**Figure S16.** Zymograms analysis for the inhibition of the mh-Tyr enzyme incubated with different concentrations of CH compound (10-1000 µg/ml).


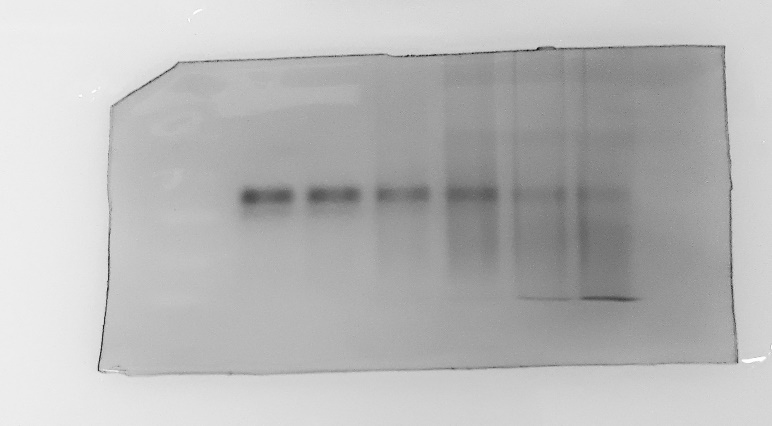


**Figure S17.** Zymograms analysis for the inhibition of the mh-Tyr enzyme incubated with different concentrations of ARB inhibitor (10-1000 µg/ml).

**S1.7. Cell viability and cell-free tyrosinase inhibition assay**

**Table S6**: Cell viability profiling for the selected compounds against human melanoma (B16F10) cell line at different concentrations.

| Stats for cell viability  (% of control) | Compounds |  | Concentration (μg/mL) | | | | | |
| --- | --- | --- | --- | --- | --- | --- | --- | --- |
|  |  |  | 0 | 10 | 50 | 100 | 500 | 1000 |
|  | C3G |  |  | 97.5 | 97.3 | 94.3 | 79.3 | 65.5 |
| AVERAGE | EC |  |  | 97.6 | 97.6 | 93.3 | 76.1 | 38.9 |
|  | CH |  | 100 | 99.0 | 98.6 | 95.7 | 82.7 | 51.3 |
|  | ARB |  |  | 96.8 | 95.7 | 95.2 | 88.8 | 85.5 |
| STDEV | C3G |  |  | 4.44 | 4.20 | 2.41 | 1.42 | 0.92 |
|  | EC |  |  | 0.27 | 1.98 | 1.61 | 3.39 | 3.18 |
|  | CH |  | 0.25 | 2.72 | 3.08 | 4.42 | 1.94 | 4.70 |
|  | ARB |  |  | 2.46 | 1.87 | 3.62 | 3.93 | 2.22 |
| *t*-test | C3G |  |  | 0.445 | 0.390 | 0.056 | 0.001 | 0.000 |
|  | EC |  |  | 0.000 | 0.174 | 0.017 | 0.006 | 0.001 |
|  | CH |  |  | 0.602 | 0.536 | 0.238 | 0.004 | 0.003 |
|  | ARB |  |  | 0.156 | 0.056 | 0.150 | 0.039 | 0.007 |

**Table S7:** Zymograms analysis for the inhibition of the murine tyrosinase enzyme incubated with selected bioactive compounds, i.e., C3G, EC, and CH, and positive control compound, viz. ARB at 100 µg/mL concentration.

| (100 μg/mL) | Control | C3G | EC | CH | ARB |
| --- | --- | --- | --- | --- | --- |
| % | 100.4 | 74.8 | 39.7 | 42.7 | 19.1 |
| STDEV | 2.23 | 1.23 | 4.06 | 4.24 | 1.76 |
| t-test | -- | 0.001 | 0.002 | 0.001 | 0.000 |


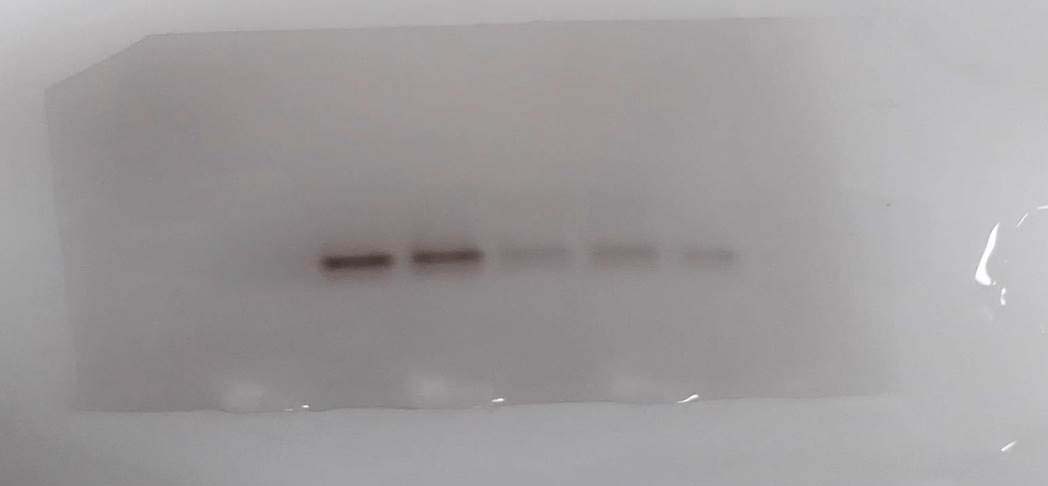


**Figure S18**. Zymogram analysis for the inhibition of the murine tyrosinase incubated with selected bioactive compounds, i.e., C3G, EC, and CH, and positive control compound, viz. ARB inhibitor at 100 µg/mL concentration.

**S1.8. Melanin content analysis**

**Table S8**: Melanin content measurement from the treated murine melanoma cells with selected bioactive compounds, i.e., C3G, EC, and CH, and positive control compound, viz. ARB inhibitor.

| (100 μg/mL) | Control | C3G | EC | CH | ARB |
| --- | --- | --- | --- | --- | --- |
| (%) | 100.9 | 81.5 | 31.6 | 33.8 | 21.8 |
| STDEV | 0.792 | 2.691 | 1.732 | 1.785 | 1.976 |
| t-test |  | 0.0063 | 0.0002 | 0.0001 | 0.0002 |

**References**

1 Schrödinger Release 2020-4: Maestro, Schrödinger, LLC, New York, NY, 2020.

2 Ismaya, W. T. *et al.* Crystal structure of Agaricus bisporus mushroom tyrosinase: identity of the tetramer subunits and interaction with tropolone. *Biochemistry* **50**, 5477-5486 (2011).

3 Bowers, K. J. *et al.* in *Proceedings of the 2006 ACM/IEEE conference on Supercomputing.* 84 (ACM).
